# Model‐Based Nanoengineered Pharmacokinetics of Iron‐Doped Copper Oxide for Nanomedical Applications

**DOI:** 10.1002/anie.201912312

**Published:** 2020-01-09

**Authors:** Hendrik Naatz, Bella B. Manshian, Carla Rios Luci, Vasiliki Tsikourkitoudi, Yiannis Deligiannakis, Johannes Birkenstock, Suman Pokhrel, Lutz Mädler, Stefaan J. Soenen

**Affiliations:** ^1^ University of Bremen Faculty of Production Engineering Badgasteiner Str. 1 28359 Bremen Germany; ^2^ Leibniz Institute for Materials Engineering IWT Badgasteiner Str. 3 28359 Bremen Germany; ^3^ NanoHealth and Optical Imaging Group KU Leuven Department of Imaging and Pathology Belgium; ^4^ Molecular Small Animal Imaging Center KU Leuven Herestraat 49 B3000 Leuven Belgium; ^5^ University of Ioannina Department of Physics Panepistimioupoli Douroutis 445110 Ioannina Greece; ^6^ Central Laboratory for Crystallography and Applied Materials University of Bremen 28359 Bremen Germany

**Keywords:** controlled pharmacokinetics, copper oxide, nanomedicine, nanoparticles, structure–release models

## Abstract

The progress in nanomedicine (NM) using nanoparticles (NPs) is mainly based on drug carriers for the delivery of classical chemotherapeutics. As low NM delivery rates limit therapeutic efficacy, an entirely different approach was investigated. A homologous series of engineered CuO NPs was designed for dual purposes (carrier and drug) with a direct chemical composition–biological functionality relationship. Model‐based dissolution kinetics of CuO NPs in the cellular interior at post‐exposure conditions were controlled through Fe‐doping for intra/extra cellular Cu^2+^ and biological outcome. Through controlled ion release and reactions taking place in the cellular interior, tumors could be treated selectively, in vitro and in vivo. Locally administered NPs enabled tumor cells apoptosis and stimulated systemic anti‐cancer immune responses. We clearly show therapeutic effects without tumor cells relapse post‐treatment with 6 % Fe‐doped CuO NPs combined with myeloid‐derived suppressor cell silencing.

## Graphical abstract

**A pharmacokinetic model**: Dissolution kinetics from iron‐doped copper oxide nanoparticles were modelled to establish structure–release relationships. With those finely tuned kinetics, a cancer treatment orthogonal to conventional chemotherapy was achieved both in vitro and in vivo. Immunization of all animals occurred, rendering them protected against tumor relapse, even upon re‐engraftment of tumor cells.

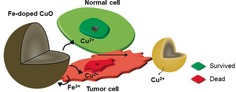

## Introduction

The wide and personalized differences between tumors, and their ability to evolve over time or acquire multidrug resistance (MDR) warrant diverse treatment options. Although small molecules and immunotherapy have made significant steps forward, they struggle with tumor adaptation and relapse, counteracting the effectiveness of these strategies.^1^ While immunotherapy applications have found their way into the clinic,^2^ their effectiveness is heterogeneous depending on the intrinsic tumor microenvironment and its level of immunosuppression.1a Nanomedicine, a high‐potential domain, faces limited clinical translation with major issues including the frequent use of classical/outdated drugs with low therapeutic efficacy against modern alternatives and a limited delivery upon intravenous administration.^3^ A review on the nanomedicine development over the past decade revealed that only 0.7 % of administered drugs are able to reach the tumor.^4^ These issues limit the use of NPs as carriers or as mediators for physical interventions.3a Hence, the translation of bio‐nanotechnology into efficient clinical trials is far from reach, although about one‐third of the US‐National Nanotechnology Initiative (NNI) budget in 2019 is proposed for the health sector.^5^

Insoluble (carrier) NPs located in the tumor microenvironment typically remain undisturbed owing to the solid extracellular matrix, dense cell‐cell packing, insufficient drainage, and prolonged particle agglomeration,^6^ while dissolving NPs can act as carrier and drug. Our decade‐long studies showed several metal oxides obtained from flame aerosol technology such as CuO, ZnO, and WO_3_ release metal ions (M^*z*+^) when they are exposed to biotic/abiotic environments.^7^ A release from metal oxides may cause strong covalent complexation between M^*z*+^ and proteins/amino acids in such dense cellular packings, subsequent complex organic–inorganic hybrid crystal precipitation in the cytoplasmic fluid, and ROS generation followed by Tier III biological cascades.^8^ Such signaling pathways, including p53 activity (oncogenic protein), play major roles in the intracellular M^*z*+^‐protein selective complexation at various levels of metabolic activity.

The investigation of anti‐cancer effects of metal oxides releasing ions, especially CuO,^9^ is incomplete/unsystematic for the following reasons: 1) The implementation of dissolution induced concepts in nanomedicine is far from reach owing to the lack of methods for adjusting pharmacokinetics, 2) the focus on studying cellular precipitation of the M^*z*+^‐cell components in crystalline form post‐complexation is missing, 3) ROS generation as a “stand‐alone” pathway for treatment is insufficient, and 4) know‐how for protecting normal peripheral cells during the proliferating cells treatment is lacking, although the local intratumoral application of therapeutic agents have been tested to reduce undesired side‐effects.^10^

**Figure 1:**
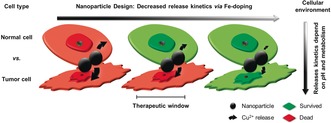
Differences in the cellular environment (pH and metabolism) affect the dissolution kinetics of metal oxides, in this case CuO NPs. Hence, we postulate that a kinetic design on the particle side, through Fe‐doping, opens a therapeutic window for cancer treatment. This requires a release that is fast enough to target cancer cells, but significantly slowed down in normal cells such that regulatory mechanisms can handle the stress.

Our own NP engineering strategy enables the fine tuning of the release of M^*z*+^ that complex with the cellular components.^11^ While the lack of a systemic effect prevents efficient anti‐cancer therapy for metastatic nodules or tumor relapse, we demonstrate the potential of targeting cancer cells and activate anti‐cancer immune responses through locally administered NPs with finely tuned pharmacokinetics. Since accelerated M^*z*+^ release is realized in nutrient‐rich (amino acids, proteins, vitamins, and/or phosphates) biological systems, intracellular release disturbs the tightly regulated metal homeostasis of tumor and peripheral cells. Owing to the differences between tumor and peripheral cells in pH and metabolism^12^ that determine the pharmacokinetics, we postulate that finely tuned Cu^2+^ release kinetics of engineered CuO nanoparticles can open a therapeutic window for cancer treatment, as schematically shown in Figure 1. If the Cu^2+^ release kinetics are too fast for cellular regulatory mechanisms, elevated copper levels cause severe damage such as proteasome inhibition leading to apoptosis of normal and cancer cells. In the reverse case, both cell types are largely unaffected owing to slow release. With the model‐based approach, our aim was to adjust the release kinetics such that predominantly cancer cells are targeted. The in vitro and in vivo studies demonstrate the potential of nanomedicine, if the efficacy of existing anti‐cancer compounds is improved.

## Results and Discussion

**Figure 2:**
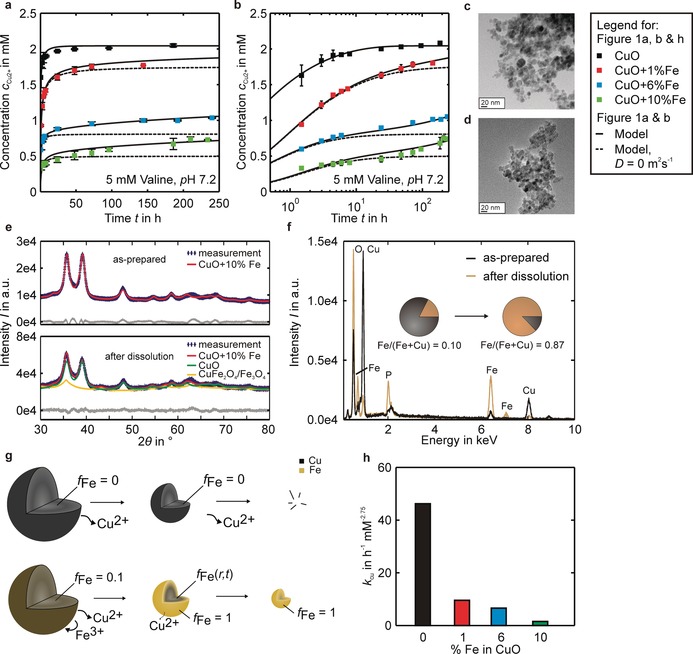
Pharmacokinetics and model for Fe‐doped CuO NPs. a) Cu^2+^ release profiles for pure, 1, 6, and 10 % Fe‐doped CuO in 5 mm valine solutions. The release behavior in 5 mm threonine, isoleucine, and serine solutions in Figure S2 a–f was similar. b) The logarithmic time scale shows the presence of two release processes for 6 and 10 % Fe‐doped CuO. The initial fast Cu^2+^ release from the CuO surface followed by a slower long‐term release from the bulk. c,d) TEM images of 10 % Fe‐doped CuO particles before and after dissolution for four weeks in 50 mm valine solution. The primary particle size and sphericity is decreased after dissolution. Similar observations were made for 6 % Fe‐doped CuO in Figure S2 a,b. e) Powder diffraction (XRD) patterns of 10 % Fe‐doped as‐prepared samples are primarily composed of the CuO phase. After dissolution, a spinel phase (CuFe_2_O_4_ or Fe_3_O_4_) formed for the 6 % (Figure S3 c) and 10 % Fe‐doped samples and the peak broadening clearly demonstrates a decreased crystallite size. f) EDX spectra (Tables S2 and S3) of the 10 % Fe‐doped samples show an increasing iron content after dissolution going beyond the release of surface available copper (Figure S5). The increased oxygen peak is likely due to the formation of the spinel. g) Differences in dissolution of pure and Fe‐doped CuO NPs, as described by the model presented in the Supporting Information. The Cu^2+^ release of pure CuO NPs results in complete dissolution. For Fe‐doped CuO the iron‐copper ratio increases during dissolution, which describes the initial release of surface available copper until an insoluble Fe‐shell forms. Further dissolution is limited by a diffusive transport of copper from the bulk to the surface. The Fe/Cu ratio becomes a function of the radial position and time, in which the dissolution stops after all copper is removed from the particle. With the three model parameters, *k*_Cu_, *k*_#,s_ and *D*, the release kinetics in (a), (b), and Figure S2 a–f (solid lines) are reasonably explained. h) Rate constants *k*_Cu_ obtained from the model decrease with increasing iron‐copper ratio *f*_Fe,0_, as the Cu−O bond length varies owing to Jahn–Teller distortion and a phase transformation from CuO to CuFe_2_O_4_ occurs during dissolution.7b

Highly crystalline pure and (1–10 %) Fe‐doped CuO NPs (ca. 10 nm, Supporting Information, Table S1) were synthesized using flame aerosol technology and characterized extensively.7b To derive differences in the ion‐release through Fe‐doping, UV/Vis spectra of particles (Figure S1 a) dispersed in model solutions (containing amino acids of the in vitro experiments) were recorded and Cu^2+^ concentration profiles were determined as described in the Supporting Information. As the particle exposure in vivo was on a long‐term basis, the dissolution kinetics of pure and Fe‐doped CuO NPs were evaluated in a 250 h‐time span in 5 mm threonine, valine, isoleucine, and serine solutions. The dissolution kinetics presented in Figure 2 a,b and Figure S2 a–f show striking differences. While the initial release rates of the Fe‐doped samples in valine solutions at *t*=0 were similar to the release from pure CuO, the rates significantly attenuated with time. On a logarithmic time‐scale in Figure 2 b, two superimposed dissolution profiles are visible for the 6 and 10 % Fe‐doped samples, a fast burst‐like release of copper in the beginning and a slow long‐term release. Particle characterization of the pure and Fe‐doped CuO NPs prior and post dissolution were carried out to identify long‐term effects. Compared to the aggregated structure of the as‐prepared spherical particles, less spherical particles were observed in TEM for 6 and 10 % Fe‐doped particles after dissolution (Figure 2 c,d and Figure S3 a,b). The observed aggregate sizes were in the same range as the values we obtained in solution using dynamic light‐scattering,7b that is, 210–664 nm. Hence, we assume a highly accessible surface during dissolution. Powder diffraction and Rietveld analysis (Figure 2 e) after dissolution showed two phases: 77(±2) mass% of original phase and 23(±1) mass% of Fe‐rich spinel phase (either CuFe_2_O_4_ or Fe_3_O_4_) for the 10 % Fe‐doped sample. For the 6 % Fe‐doped sample, we found 42(±2) mass% of original phase and 60(±2) mass% of spinel phase (Figure S3 c). Accordingly, the Fe‐enrichment and the dissolution of original phase are higher for the Fe‐doped phase with lower initial Fe/(Fe+Cu) ratio. Rietveld analysis only considers the crystalline parts of the samples. The total composition measured by EDX spectroscopy of the as‐prepared 10 % Fe‐doped sample yielded Fe/(Fe+Cu)=10.4 at % (close to the nominal value), increasing to 87.3 at % after dissolution (Figure 2 f, Tables S2 and S3). Therefore, far more than the surface‐available copper is dissolved on the long‐term. While our earlier cyclic voltammetry7b results showed the formation of CuFe_2_O_4_, these new findings suggest a transformation from CuO via CuFe_2_O_4_ to Fe_3_O_4_ during dissolution, together with the formation of an amorphous Fe‐O (Cu‐free) phase.

Based on kinetics and particle characterization, a model was developed to understand and predict the differences in Cu^2+^ release from pure and Fe‐doped CuO NPs (Figure 2 g). The model includes copper surface release, a variable iron/copper ratio and solid‐state diffusion for bulk copper release. Model assumptions for the dissolution of pure CuO are as follows: 1) A rate constant *k*_Cu_ is sufficient to describe the rate of copper release $\frac{dc_{{Cu}^{2+}}}{dt}$ , 2) the rate is proportional to the surface copper concentration $c_{Cu,s}^{m}$ , and free amino acids $c_{AA}^{n}$ , where both the proportionalities agree with the power law with partial dissolution orders *m* and *n*, respectively,^13^ 3) Cu^2+^ and amino acids complex in the ratio of 1:2 at physiological pH,^14^ evidenced through single‐crystal X‐ray diffraction (SC‐XRD) of light blue precipitates in glutamine. The SC‐XRD analysis confirmed Cu^II^‐l‐glutamine (Cu[NH_2_CO_2_CH(CH_2_)_2_CONH_2_]_2_) (space group *C2*). Within standard uncertainties, bond distances and lattice parameters are in agreement with the structure reported^15^ (Figure S3 d and Table S4). 4) The surface copper concentration is proportional to the active surface area (mathematical details of the model in Supporting Information). The resulting model reasonably describes release kinetics of pure CuO NPs with *m*=1.75 and *n*=2, when the dissolution rate constant *k*_Cu_ is independent of the initial CuO concentration, that is, $\frac{{dk}_{Cu}}{{dc}_{CuO,0}}\approx 0$ , and the difference between experiment and model is expressed by a minimum in the mean square error (Figure S4 a–c). Rate constants derived from the dissolution profiles clearly illustrate a selective binding for the amino acids (Figure S4 d,e). To model the two‐step dissolution Fe‐doped CuO, the material composition was considered assuming the following: 1) CuFe_2_O_4_ and Fe_3_O_4_ formation (Fe redistributes on the particle surface during Cu^2+^ release such that the iron/copper surface ratio $f_{Fe,s}=\frac{{Fe}_{s}}{{Cu}_{s}+{Fe}_{s}}$ increases with dissolution until all the surface copper is released leaving Fe at the surface (*f*_Fe,s_=1). The absence of simultaneous Fe^3+^ release was evidenced through a spot test using potassium hexacyanoferrate(III) (Figure S4 f). 2) Without solid‐state diffusion (*D*=0 m^2^ s^−1^), dissolution would stop at *f*_Fe,s_=1 (dashed lines in Figure 2 a,b), but the long‐term release goes beyond this limit, including copper from the core region (Figure S5). To implement solid‐state diffusion, Fick's second law was solved with an explicit numerical scheme using radial symmetry. The non‐linear moving‐boundary condition was derived from a global mass balance (Supporting Information) resulting in a two‐step dissolution process, but without satisfying conservation of mass. To obey conservation of mass, the solution was split into fast release of surface‐available copper, followed by a diffusion‐limited dissolution assuming *f*_Fe_(*r*,0)=*f*_Fe,0_ in the bulk and *f*_Fe,s_=1 at the surface. Model details and validation of mass conservation are presented in the Supporting Information and Table S5. Superimposing both solutions enabled a reasonable description of Fe‐doped CuO NPs dissolution (solid lines in Figure 2 a,b) with the three fit parameters, rate constant *k*_Cu_, atomic surface density *k*_#,s_ determining the rate of change in the iron‐copper ratio, and diffusion coefficient *D*. While dissolution is driven by the surface properties, increasing Fe‐doping reduces the rate constants *k*_Cu_ in all amino acid solutions (Figure 2 h and Table S1) owing to strong Jahn–Teller distortion, that is, different apical and planar Cu−O bond lengths stabilizing the particles.7b The dose from the burst‐like release (dashed lines) is determined by the initial particle diameter *d*_0_ and iron/copper ratio *f*_Fe,0_. Still, a complete release of copper is feasible and enables a long‐term release owing to solid‐state diffusion. Diffusion coefficients determined in this work are on the order of 10^−27^ m^2^ s^−1^. Values for diffusion in metal oxides are commonly reported at higher temperatures (>500 °C), such that the best comparison with literature is an extrapolation to room temperature using the Arrhenius equation. However, this approximation underestimates the diffusion coefficients because extrinsic factors such as impurities and defects govern diffusion at room temperature.^16^ Hence, solid‐state diffusion at room temperature is commonly neglected, but obviously plays a role at the nanoscale. Fe‐doping enables precisely controlled Cu^2+^ release from CuO NPs with a prolonged release for a lasting treatment, with *d*_0_ and *f*_Fe,0_ being key parameter in designing the pharmacokinetics of CuO as nanomedicine.

To verify our findings even in more complex biological environments, electron paramagnetic resonance (EPR) spectra were recorded in a growth medium (RPMI for comparison with7b). While the spectra for pure CuO showed strong exchange‐coupling of the crystalline material, Fe‐doped samples showed Fe islands (CuFe_2_O_4_/Fe_3_O_4_,) on the surface (Figure S6 a). In the growth medium at pH 7, a release of 65±5 % of the total Cu^2+^ from pure CuO within 10 min was observed, while the release was drastically decreased in the presence of Fe. While 8 % of the total Cu^2+^ was released from the 10 % Fe‐doped CuO during the same time span and only a very small fraction of Fe atoms (<2 %) was released (evidenced by a line at *g*=4.3, an EPR fingerprint region for Fe^3+^, Figure S6 c,d). A small fraction of Cu^2+^ is re‐adsorbed on the particle surface without complexation with protein, as evidenced by EPR linewidths analysis.^17^ Most striking, all released Cu^2+^ atoms are immediately bound (broad peak at *g*≈4 indicates a Cu–Cu coupling owing to the proximity of the Cu atoms during complexation) with proteins and/or amino acids present in the medium (Figure S6 d) followed by crystallization/precipitation.

**Figure 3:**
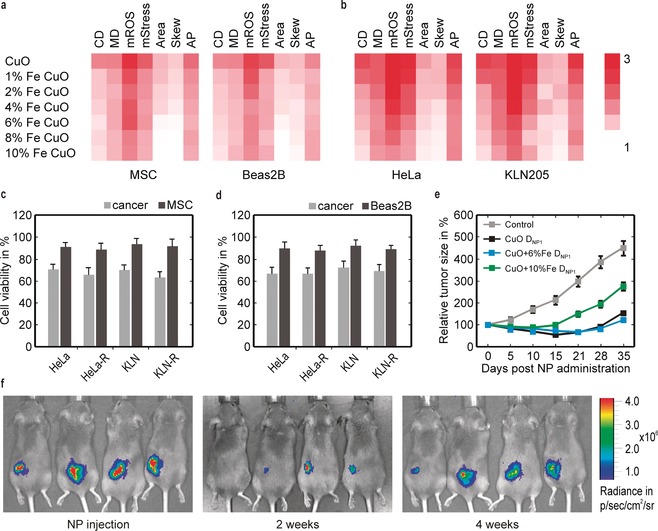
Therapeutic efficacy of Fe‐doped CuO NPs against different cancer types. a,b) High content imaging data for the indicated cell types exposed to the different NPs at 12.5 μg mL^−1^. The fold‐difference compared to untreated control cells is indicated in color‐code for CD=cell death, MD=membrane damage, mROS=mitochondrial ROS, mStress=mitochondrial stress, Area=cell size, Skew=cell skewness, and AP=autophagy. Histograms presenting the cellular parameters indicated for both normal cells and cancer cells (both wild‐type and resistant) grown in co‐culture experiments for c) MSC and d) Beas2B cells and exposed to 6 % Fe‐doped CuO at 12.5 μg mL^−1^. e) Relative luminescence signals for firefly luciferase‐expressing KLN 205 cells grafted subcutaneously in syngeneic DBA/2 mice and treated with CuO, 6 % Fe‐doped CuO, 10 % Fe‐doped CuO given at *D*_NP1_=125 μg/mouse. f) Representative luminescence images of DBA/2 mice bearing firefly luciferase‐expressing KLN 205 cells treated with 6 % Fe‐doped CuO (*D*_NP3_=225 μg/mouse), 0 (left), 2 (middle), and 4 weeks (right) after NP injection. The data are presented as mean ±SD (number of animals *n*=8).

Finally, the CuO‐based particle dissolution kinetics were investigated in vitro for biological response in two normal (mesenchymal stem cells (MSC) and lung cells (Beas‐2B)) and two cancerous cell types (cervical (HeLa) and lung adenocarcinoma (KLN‐205)) using high‐content imaging. A clear inverse correlation between cell response and Fe‐doping was observed for all cell types (Figure 3 a,b). A predominant increase in ROS generation and cell death for both cancer cell types suggested a selective response of CuO NPs. Under conditions in which normal cells were statistically unaffected, the maximal response against cancer cells was observed for 6 % Fe‐doped CuO NPs at 12.5 μg mL^−1^. Upon induction of drug resistance, the efficacy of the NPs remained unaffected (Figure 3 c,d and Figure S7 a,b), indicating the NP efficacy is independent of the acquired therapeutic resistance.^18^

In a syngeneic subcutaneous KLN‐205 animal model, 6 % Fe‐doped CuO NPs showed more potency for tumor therapy than 10 % Fe‐doped CuO NP at the same dosage (125 μg kg^−1^ bodyweight (bw)) (Figure 3 e,f). Pure CuO and 6 % Fe‐doped CuO had similar effects, but pure CuO affected animal weight and led to the premature death of some animals (Figure S7 c). For 6 % Fe‐doped CuO NPs, the dose was further increased to at least 225 μg kg^−1^ bw without any gross effects on animal wellbeing. However, even at higher doses and repeated administration (2nd dose at 15 days), the NPs alone resulted in incomplete tumor remission (Figure S7 d), requiring the parallel induction of anti‐cancer immunity for potent therapeutic efficacy.

**Figure 4:**
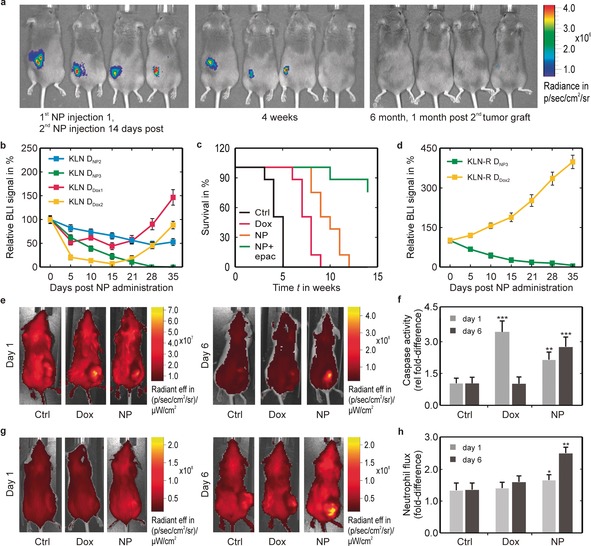
Therapeutic efficacy of combined treatment with Fe‐doped CuO NPs and epacadostat against different cancer types. a) Representative luminescence images (BLI) of DBA/2 mice bearing firefly luciferase‐expressing KLN 205 cells (KLN) treated with 6 % Fe‐doped CuO (*D*_NP3_=225 μg/mouse, 2^nd^ injection after 14 days) and epacadostat (left, NP injection; middle, 4 weeks post‐injection; right, 6 month post‐injection and 1 month post‐second tumor graft). After tumor remission, animals received a second graft of luminescent KLN 205, but the animals remained free from tumor relapse for up to 6 months. b) Relative bioluminescence imaging (BLI) signals for firefly luciferase‐expressing KLN 205 cells grafted subcutaneously in syngeneic DBA/2 mice and treated with 6 % Fe‐doped CuO (*D*_NP2_=175 μg/mouse or *D*_NP3_=225 μg/mouse) + epacadostat or Doxorubicin (*D*_Dox1_=2 μmol kg^−1^ or *D*_Dox2_=5 μmol kg^−1^) + epacadostat. c) Kaplan–Meier survival curves for DBA/2 mice bearing KLN 205 tumors and treated with saline (control), doxorubicin (*D*_Dox2_=5 μmol kg^−1^) or 6 % Fe‐doped CuO (*D*_NP3_=225 μg mL^−1^) with or without + epacadostat. d) Relative luminescence signals for firefly luciferase‐expressing KLN 205 cells resistant against doxorubicin (KLN‐R) and grafted subcutaneously in syngeneic DBA/2 mice and treated with 6 % Fe‐doped CuO (*D*_NP3_=225 μg mL^−1^) + epacadostat or Doxorubicin (*D*_Dox2_=5 μmol kg^−1^) + epacadostat. Data are presented as mean ± SD (*n*=8). e) Representative fluorescence images of Balb/c mice with syngeneic CT26 model treated with a fluorescent pan‐caspase probe. Animals were treated with either saline, doxorubicin (*D*_Dox2_=5 μmol kg^−1^) + epacadostat or 6 % Fe‐doped CuO (*D*_NP3_=225 μg mL^−1^) + epacadostat. Images are shown for 1 day (left) and 6 days (right) post‐treatment. f) Quantification of fluorescence signal of pan‐caspase treated mice as described in (e). Data are presented as mean ± SD (*n*=4). g) Representative fluorescence images of Balb/c mice with syngeneic CT26 model treated with a fluorescent neutrophile‐specific peptide. Animals were treated with either saline, doxorubicin (*D*_Dox2_=5 μmol kg^−1^) + epacadostat or 6 % Fe‐doped CuO (*D*_NP3_=225 μg mL^−1^) + epacadostat. Images are shown for 1 day (left) and 6 days (right) post‐treatment. h) Quantification of fluorescence signal of pan‐caspase treated mice as described in (g). Data are presented as mean ± SD (*n*=4). The number of asterisks indicate the level of significance, where: *=*p*<0.05, **=*p*<0.01 and ***=*p*<0.001.

Checkpoint inhibitors are prone to causing off‐target effects, and therapies have focused on inhibition of immunosuppression, for example, indoleamine 2,3‐dioxygenase (IDO1) inhibitors,^19^ preventing immunosuppression through myeloid‐derived suppressor cells (MDSCs).^20^ Epacadostat, a strong IDO1 inhibitor showed good preclinical results, but failed in a phase III clinical trial together with immune checkpoint inhibitors in metastatic melanoma. One major reason was the choice of the tumor model, for which checkpoint inhibition already has a good effect as a stand‐alone method, and neither of both methods (checkpoint inhibitor or anti‐cancer agent) may intrinsically result in an activation of the immune system.^21^ In this study, we look at the effect of epacadostat and the 6 % Fe‐doped CuO NPs for their combined therapy. The combination of epacadostat and 6 % Fe‐doped CuO NPs resulted in enhanced therapeutic efficacy and complete tumor remission, in contrast to animals treated with epacadostat and doxorubicin (Figure 4 a–c). Repeated NP exposure at high dose (225 μg/mouse) showed absence of any clinical effects on blood biochemistry (Figure S8) while histological evaluation of major organs even showed the absence of macroscopic effects. Similar to the cellular studies, the epacadostat and 6 % Fe‐doped CuO were equally effective against drug‐resistant tumors (Figure 4 d). Animals bearing luminescent KLN‐205 tumors, successfully treated with the NPs and kept for further 12 weeks, showed absence of tumor relapse. The re‐engraftment of KLN‐205 cells in all these animals failed to produce growing tumors indicating a potent anti‐cancer vaccination incurred by the parallel treatment with epacadostat and NPs.

To demonstrate the applicability of NP dissolution‐induced medicine, a syngeneic subcutaneous model of CT26 (colon) tumors in Balb/c mice were used. While the therapeutic efficacy of dissolving NPs was increased in immune‐competent animal models,^22^ we mainly focused on the role of the immune system. The CT26 cells were directly treated with the CuO NPs under identical conditions as the KLN‐205 cells (Figure 4 e). As luminescent reporter genes can induce undesired alterations in cellular immunogenicity, untransformed wild‐type cells were used to probe for active caspase‐3 (triggers apoptosis).^23^ Results showed NPs induced responses at a later stage than doxorubicin (Figure 4 f), which is in line with the data on tumor growth and our kinetic predictions. To see first signs of inflammation, a Cy7‐labeled neutrophil‐specific hexapeptide was used, demonstrating a clear immune activation through influx of neutrophils upon tumor exposure to NPs and epacadostat compared to doxorubicin and epacadostat (Figure 4 g,h). NP‐driven anticancer immunity has been found to be due to NLRP3 inflammasome activation,^24^ and the potent anticancer effect suggests that the cancer cell‐death mechanism is immunogenic.^25^ A gradual long‐term release of Cu^2+^ ions over time together with ROS generation reaches toxic levels offering vaccination‐like effect for anti‐tumoral immune activation.^26^ To confirm, tumors were isolated from treated animals, revealing an increased influx of different immune cells, activation of cytotoxic (CD8+) T cells and natural killer cells, confirming the propensity of the NPs to elicit a local antitumor immune response (Figures S9 and S10). Combined with the lack of growth upon tumor cell re‐challenge, this suggests a potent anti‐cancer immune activation.

**Figure 5:**
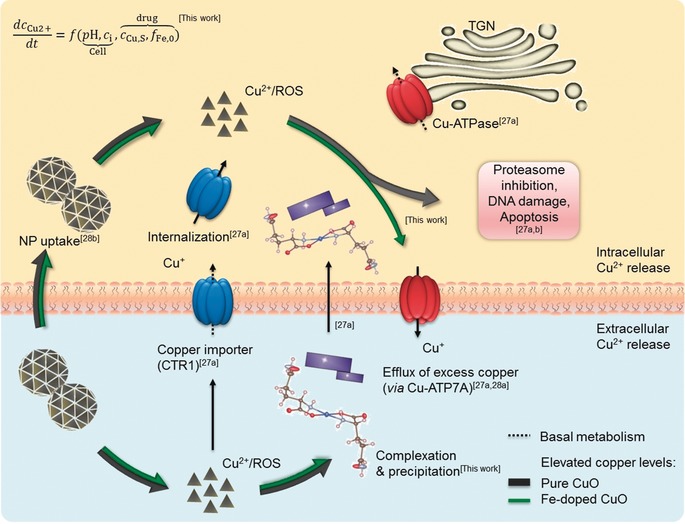
Copper homeostasis and regulatory mechanisms including extra‐ and intracellular dissolution of pure and Fe‐doped CuO NPs. For the basal metabolism involving the secretory pathway, copper uptake is regulated by copper importing proteins, for example, CTR1. In case of elevated extracellular copper levels, CTR1 is internalized, while intracellular excess copper is excreted by ATPase. When overwhelmingly large [Cu^2+^] is present in the cell (case of pure CuO), the Cu‐ATPase fails to function (all the cells die). When it is too low (10 % Fe‐doping), the ions are easily reduced and transported (no effect). In the third case (6 % Fe‐doping) when the Cu^2+^ release is controlled, the complexation with the cellular constituents and the Cu^2+^/Cu^+^ redox balance can precisely regulate the target cell apoptosis without Cu^2+^ diffusing to the normal cells. Our findings are embedded in the regulatory mechanisms of copper homeostasis proposed in the literature,^27^, ^28^ see references in the figure.

A strict regulation of copper homeostasis (Figure 5) is necessary to avoid adverse effects connected to elevated intracellular copper levels, such as ROS generation, DNA damage, proteasome inhibition, and induction of apoptosis. Regulatory mechanisms involve uptake through copper importers (e.g., CTR1) or complexes and excretion of excess copper by copper ATPase (e.g., Cu‐ATP7A). At elevated copper levels, CRT1 is internalized and ATPase relocates from the trans‐Golgi network (TGN) to the plasma membrane to excrete excess copper, instead of the basal delivery function to the secretory pathway.^27^ While CTR1 internalization reduces uptake of extracellular dissolved copper (Cu^+^) in the tumor microenvironment, intracellular dissolution of CuO NPs (Cu^2+^) after uptake is unhindered and results in redox imbalance as well as intrinsic ROS generation (lattice oxygen). Subsequent reduction of Cu^2+^ increases oxidative stress. By finely tuning the release kinetics of CuO NPs, we aimed to increase the copper bioavailability and parallel ROS generation in cancer cells. In the present study we have three different scenarios, very fast, very slow, and intermediate Cu^2+^ release kinetics of pure CuO, 10 % and 6 % Fe‐doped CuO (Figure 2 a,b), respectively.

When pure CuO is used in the different cell and animal models, the Cu‐ATPases start functioning at once owing to the high concentration of Cu^2+^ entering the cellular interior. The release will be much higher than Cu^2+^‐transport through Cu‐ATPases for copper homeostasis in maleficent and peripheral cells. The malfunction of the Cu‐ATPases and the cells with high Cu^2+^ will trigger Cu^2+^/Cu^+^ redox imbalance leading to cell death (reduced body weight and animal death). In the reverse case, slow [Cu^2+^] released by 10 % Fe‐doped CuO is conveniently transported to target sites and excreted through functional Cu‐ATPases maintaining the Cu^2+^/Cu^+^ redox balance. In the third case, the difference in the finely tuned release kinetics between tumor cells (more acidic environment pH↓ and higher metabolism *c*_i_↑) and peripheral cells was sufficient to open a therapeutic window. The proposed mechanism is in line with the “lysosomal Trojan horse effect”, describing the ability of dissolving NPs to act as cytotoxic agents when present in the low pH of the endosomes.28b The specificity of the NPs towards cancer cells remains somewhat elusive. Different mechanisms have been described, linking NP toxicity to functional p53 levels, a commonly affected oncogene.^29^ However, p53‐related mutations are absent in CT26 cells, as used in this study, cancelling this hypothesis.^30^ Alternatively, one mechanism may lie in the intrinsic ability of the NPs to cause mitochondrial damage. Mitochondrial metabolism has been shown to be a major player in the formation of neoplasms and affects therapeutic resistance.^31^ Flexibility of cancer cells in tuning their mitochondrial metabolism can therefore drive resistance but can also render the cells sensitive to targeted therapy. In this case, the influx of excess Cu^2+^ along with elevated mitochondrial metabolism may preferentially affect cancer cells. From a simplistic point of view, Cu ions are involved in scavenging oxidative stress, but excess of Cu ions and elevated ROS can synergistically result in toxicity. For normal cells with lower metabolic rates, the additional ROS can result in transient cellular damage, allowing the cells to recover, while for cancer cells, the higher levels can exceed toxic thresholds, resulting in an effective tumor therapeutic. This emphasizes the need for a strictly controlled dissolution kinetics to avoid exceeding toxic levels in normal cells, while tipping the balance towards cell death in cancer cells. Additionally, targeting mitochondrial metabolism can be difficult to direct, as many types of immune cells, including cytotoxic T lymphocytes (CD8+ T cells) share many of the same characteristics.^31^ The advantage of the nanoformulations and the requirement for lysosomal processing, which is far less suitable in T cells, makes this therapeutic agent intrinsically proficient against tumor cells. While peripheral cells seem to maintain copper homeostasis due to slower release kinetics, [Cu^2+^] remaining in the proliferating cells after the transportation and excretion by Cu‐ATPases was sufficient for tumor remission, clearly validating the statement by Robinson et al. “Copper is vital to most cells, but too much is lethal”.28a

## Conclusion

In this work, we precisely controlled the dissolution kinetics of CuO NPs in biological environments through iron‐doping. The incorporation of iron resulted in a two‐step dissolution with an initial fast release followed by a slow long‐term release. The implemented pharmacokinetic model predicts the experimentally observed behavior and provides significant insights into structure–release relationships during the dissolution of metal oxide nanoparticles. By controlling the pharmacokinetics, a cancer‐treatment orthogonal to conventional approaches such as chemotherapy was achieved in vitro and in vivo. In a combined treatment with a clinically approved agent against immunosuppression (IDO1), the Fe‐doped CuO NPs were tested in vivo, resulting in complete tumor remission in multiple syngeneic subcutaneous mouse models. Most interestingly, the NP‐mediated degradation together with chemical lifting of immune inhibition resulted in the generation of a systemic immune effect and the immunization of all animals, rendering them protected against tumor relapse, even upon an additional tumor cell engraftment. We strongly believe that our interdisciplinary approach is viable, economic, safe, and therefore a supplement to conventional treatment that might induce a paradigm shift in the near future.

## Conflict of interest

The authors declare no conflict of interest.

## Supporting information

As a service to our authors and readers, this journal provides supporting information supplied by the authors. Such materials are peer reviewed and may be re‐organized for online delivery, but are not copy‐edited or typeset. Technical support issues arising from supporting information (other than missing files) should be addressed to the authors.

Supplementary
