# Supplementary material for: Model‐Based Nanoengineered Pharmacokinetics of Iron‐Doped Copper Oxide for Nanomedical Applications
Source: Angew Chem Int Ed Engl. 2020 Jan 9;59(5):1828–36. doi: 10.1002/anie.201912312 (PMC7004194; doi:10.1002/anie.201912312)
Supplement: Supplementary file 1 — Supplementary [file ANIE-59-1828-s001.pdf]

Angewandte Chemie
Eine Zeitschrift der Gesellschaft Deutscher Chemiker

Supporting Information

Model-Based Nanoengineered Pharmacokinetics of Iron-Doped Copper Oxide for Nanomedical Applications

Hendrik Naatz, Bella B. Manshian, Carla Rios Luci, Vasiliki Tsikourkitoudi, Yiannis Deligiannakis, Johannes Birkenstock, Suman Pokhrel, Lutz Mädler, and Stefaan J. Soenen**

anie_201912312_sm_miscellaneous_information.pdf

Supporting Information

Abstract: The progress in nanomedicine (NM) using nanoparticles (NPs) is mainly based on drug carriers for the delivery of classical chemotherapeutics. As low NM delivery rates limit therapeutic efficacy, an entirely different approach was investigated. A homologous series of engineered CuO NPs was designed for dual purposes (carrier and drug) with a direct chemical composition-biological functionality relationship. Model-based dissolution kinetics of CuO NPs in the cellular interior at post-exposure conditions were controlled through Fe-doping for intra/extra cellular Cu^{2+} and biological outcome. Through controlled ion release and reactions taking place in the cellular interior, tumors could be treated selectively, *in vitro* and *in vivo*. Locally administered NPs enabled tumor cells apoptosis and stimulated systemic anti-cancer immune responses. We clearly show therapeutic effects without tumor cells relapse post-treatment with 6% Fe-doped CuO NPs combined with myeloid-derived suppressor cell silencing.

DOI: 10.1002/anie.201912312

Table of Contents

Experimental Procedures 2
Synthesis of pure and Fe-doped CuO NPs 2
UV-visible measurements 2
Kinetic model 2
XRD, TEM and EDX measurements 4
Electron paramagnetic resonance spectroscopy 5
Cell Cultures 5
Cell-nanoparticle interaction studies 5
Generation of Doxorubicin-resistant cancer cells 5
Cell viability in co-culture experiments 5
Mice experiments 6
Monitoring of tumor growth 6
Statistical analysis 6
High content cell analysis 6
ImageStream analysis 7
Blood biochemistry analysis 8
Monitoring of neutrophil influx 8
Monitoring of *in-vivo* caspase activity 8
Tissue immunofluorescence 8
Results 9
References 21

Experimental Procedures

Synthesis of pure and Fe-doped CuO NPs. Flame aerosol technique was used to synthesize pure and/or Fe-doped CuO NPs. The metal-organic precursors copper napthenate (8% Fe, Strem Chemicals) iron napthenate (12% Fe by metal, Strem Chemicals) were dissolved in xylene (Strem Chemicals, 99.95 pure) and the required amount of iron napthenate was mixed with the copper napthenate solution before combustion ([1] for details). The flame aerosol process for NPs synthesis is described elsewhere[2].

UV-visible measurements. 5 mM pure or Fe-doped CuO NP suspensions in Di-water and 10 mM amino acid (Sigma-Aldrich) solutions, buffered with 3.7 g/mL sodium bicarbonate (Sigma-Aldrich), were separately prepared. Directly before the measurement, the required ratios of both solutions were mixed in a 20 mL glass bottle, *e.g.* 1:1 for 2.5 mM CuO in 5 mM amino acid solution. For each measurement a portion of 2 mL was transferred to a 10 mm cuvette and the absorbance spectra were recorded with a Shimadzu UV-visible spectrophotometer UV-2600 equipped with an ISR-2600Plus integrating sphere, without a reference cuvette (air as reference). To keep the particles in suspension, a magnetic stirrer (Hellma Analytics) was used at 400 RPM for investigation of the initial release kinetics in Figure S1. For the long-term experiments in Figure 2, the suspensions were ultra-sonicated (Retsch) for 10 seconds every working day and prior to each measurement. Known concentrations of $CuCl_2$ (Sigma-Aldrich) were used to calibrate the signal intensities. pH values were adjusted with HCl and NaOH and monitored with indicator stripes (Chemsolute). For each dissolution profile, two series were recorded and mean, min and max values were calculated.

Kinetic model. Based on assumptions in the manuscript, the dissolution rate of pure CuO NPs is proportional to a rate constant k_{Cu}, the surface copper concentration $c_{\mathrm{Cu,s}}^{m}$ and the free amino acid concentration c_{AA}^{n}, where m and n denote the partial reaction orders. Cu^{2+} is released to the same extend as CuO dissolves and lattice oxygen is assumed to react simultaneously to form reactive oxygen species (ROS). The change in free amino acid concentration is two times faster at neutral pH[1], *i.e.* $Cu^{2+} + 2L^{-} \rightarrow CuL_2$. Concentration profiles for pure CuO are obtained from the solution of the ordinary differential equations (ODEs) in Eqs. (1)-(3).

$$\frac{dc_{\mathrm{CuO}}}{dt} = -k_{\mathrm{Cu}} c_{\mathrm{Cu,s}}^{m} c_{\mathrm{AA}}^{n} \tag{1}$$

$$\frac{dc_{\mathrm{Cu2+}}}{dt} = k_{\mathrm{Cu}} c_{\mathrm{Cu,s}}^{m} c_{\mathrm{AA}}^{n} \tag{2}$$

$$\frac{dc_{\mathrm{AA}}}{dt} = -2\, k_{\mathrm{Cu}} c_{\mathrm{Cu,s}}^{m} c_{\mathrm{AA}}^{n} \tag{3}$$

The surface copper concentration $c_{\mathrm{Cu,s}}$ is proportional to the shrinking surface area of the particles and calculated according to Eq. (4) from the atomic number density $k_{\#,v} = \frac{\rho_{\mathrm{CuO}} N_{\mathrm{A}}}{M_{\mathrm{CuO}}}$ and the surface shell volume $a \times \Delta s$ occupied by the atomic layer in contact with solution (Figure S5a). The shell thickness Δs was estimated from the atomic radius of Cu^{2+} (0.073 nm[4]). Multiplying Δs and $k_{\#,v}$ results in a new constant $k_{\#,s}$, which is the copper concentration per unit surface area. For pure CuO, $k_{\#,s}$ is indistinguishable from the rate constant k_{Cu}, but for Fe-doped CuO it determines the rate of change in surface concentration and the amount of surface available copper (Figure 5Sb-d).

$$c_{\mathrm{Cu,s}} = k_{\#,v}(a \times \Delta s) = k_{\#,s} a \tag{4}$$

The particle surface area a and radius r decrease proportional to the volume v. Considering n_{p} spherical particles, changes are given by Eq. (5)-(7).

$$\frac{dv}{dt} = \frac{dc_{\mathrm{CuO}}}{dt} \frac{M_{\mathrm{CuO}}}{\rho_{\mathrm{CuO}}} \tag{5}$$

$$\frac{da}{dt} = \frac{da}{dv} \frac{dv}{dt} \tag{6}$$

$$\frac{dr}{dt} = \frac{1}{n_{\mathrm{p}}} \frac{dr}{dv} \frac{dv}{dt} \tag{7}$$

The system of differential Eqs. (1)-(7) was solved in Matlab using ODE45 ('RelTol' = 1e-10 and 'AbsTol' = 1e-12) with the initial conditions $c_{\mathrm{CuO,0}}$[2], $c_{\mathrm{Cu2+,0}} = 0\ mM$, $c_{\mathrm{AA,0}} = 5\ mM$ and $r_0 = 0.5 d_{\mathrm{BET}}$ (taken from[5], Table S1). The initial volume $v_0 = c_{\mathrm{CuO,0}} \frac{M_{\mathrm{CuO}}}{\rho_{\mathrm{CuO}}}$, the

1 Under more acidic conditions of many cancer cells, the reaction mechanism alters [3]. Similarly, the reported crystallization is strongly pH selective.
2 The nominal amount of 2.5 mM resulted in less CuO in solution due to humidity accounting for approximately 15 wt.-% of the weighted mass.

initial surface area $a_0 = \frac{3v_0}{r_0}$ and the number of particles in solution $n_\mathrm{p} = \frac{v_0}{\frac{4}{3}\pi r_0^3}$ were calculated accordingly[3]. The partial reaction orders m and n were determined such that the rate constant was independent of the particle concentrations and the mean square error between model and experiment was minimized (Figure S4b-c). For the Cu^{2+} release from Fe-doped CuO NPs, the bulk iron-copper ratio $f_\mathrm{Fe,0} = \frac{\mathrm{Fe_0}}{(\mathrm{Fe_0+Cu_0})}$ was implemented. Validity of the rate law derived for pure CuO, *i.e.* partial reaction orders $m = 1.75$ and $n = 2$, is similarly assumed for Fe-doped CuO. During dissolution, copper is released, but iron remains at the particle such that the surface iron-copper ratio $f_\mathrm{Fe,s}$ increases. If a certain amount of copper $c_\mathrm{Cu2+}$ is released, $c_\mathrm{Fe,s} = c_\mathrm{Cu2+}\frac{\mathrm{Fe_0}}{(\mathrm{Fe_0+Cu_0})}N_\mathrm{A}$ redistributes on surface according to Eq. (8), where N_A is the Avogadro constant[4].

$$\frac{dc_\mathrm{Fe,s}}{dt} = \frac{dc_\mathrm{Cu2+}}{dt}\frac{\mathrm{Fe_0}}{(\mathrm{Fe_0} + \mathrm{Cu_0})}N_\mathrm{A} \qquad (8)$$

The number of copper surface atoms $c_\mathrm{Cu,s}$ (Eq. (9)) at any time is given by the copper-iron surface ratio $f_\mathrm{Cu,s} = \frac{\mathrm{Cu}_S}{(\mathrm{Fe}_S+\mathrm{Cu}_S)}$, multiplied with the atomic number density $k_{\#,v}$ and the surface shell volume $a \times \Delta s$, where $k_{\#,s} = k_{\#,v} \times \Delta s$.

$$c_\mathrm{Cu,s} = k_{\#,s}a\frac{\mathrm{Cu_s}}{(\mathrm{Cu_s} + \mathrm{Fe_s})} \Leftrightarrow c_\mathrm{Cu,s} = k_{\#,s}a - c_\mathrm{Fe,s} \qquad (9)$$

Applying the chain rule, Eq. (10) gives the decrease of the copper ions at the surface.

$$\frac{dc_\mathrm{Cu,s}}{dt} = k_{\#,s}\frac{da}{dt} - \frac{dc_\mathrm{Cu2+}}{dt}f_\mathrm{Fe,0}N_\mathrm{A} \qquad (10)$$

$\frac{da}{dt}$ is given by Eq. (6) and $\frac{dc_\mathrm{Cu2+}}{dt}$ by Eq. (2), respectively. With the increasing surface iron-copper ratio $f_\mathrm{Fe,s}$, the Cu^{2+} release decreases gradually until all surface available copper is in solution, *i.e.* $f_\mathrm{Fe,s} = 1$. The dissolution stops at this point, reaching the final particle diameter $d_\mathrm{Fe,s=1}$ (Figure S5d). Eqs. (8) and (10) were added to the DGL-system in Eqs. (1)-(7) and solved analogously with the initial Fe and Cu surface concentrations determined from the bulk ratio $f_\mathrm{Fe,0}$. To include the release of copper from the CuO+Fe core region (Figure S5b-d), solid state diffusion was implemented in the model using Fick's second law in spherical coordinates (Eq. (11)) with a symmetry boundary condition at $r = 0$ (Eq. 12) and the moving boundary condition at the particle-solution interface $r = R(t)$ (Eq. 13).

$$\frac{\delta c_\mathrm{Cu}(r,t)}{\delta t} = \frac{1}{r^2}\frac{\delta}{\delta r}\left[r^2 D\frac{\delta c_\mathrm{Cu}(r,t)}{\delta r}\right], \qquad 0 < r < R(t) \qquad (11)$$

$$\frac{\delta c_{Cu}(0,t)}{\delta r} = 0 \qquad (12)$$

$$D\frac{\delta c_\mathrm{Cu}(R(t),t)}{\delta r} = [c_\mathrm{P} - c_\mathrm{Cu}(R(t),t)]\frac{dR(t)}{dt} \qquad (13)$$

The moving boundary condition Eq. (13) describes the flux of iron $c_\mathrm{Fe}(R(t),t)\frac{dv}{dt} = (c_\mathrm{p} - c_\mathrm{Cu}(R(t),t))\frac{dv}{dt}$ through the shrinking surface, if a certain amount of copper $c_\mathrm{Cu}(R(t),t)\frac{dv}{dt}$ is released. $c_\mathrm{P} = k_{\#,v}$ is atomic number density. Eq. (13) can be derived from a copper mass balance for one particle in Eq. (14). The left-hand side corresponds to the total copper in the initial particle m_i. The first term on the right-hand side is the remaining copper after a certain time $m_\mathrm{P}(t)$ and the second term is the total amount of dissolved copper $m_\mathrm{D}(t)$ during the same time (Figure S5e). $m_\mathrm{D}(t)$ equals the shell volume removed from the particle, multiplied by the total concentration of atoms in this volume c_P, since the volume removed during dissolution is only the one of the copper phase.

$$\underbrace{\frac{4}{3}\pi R_0^3 c_\mathrm{Cu,0}}_{m_i=const} = \underbrace{\int_0^{R(t)} 4\pi r^2 c_\mathrm{Cu}(r,t)dr}_{m_\mathrm{P}(t)} + \underbrace{\frac{4}{3}\pi(R_0^3 - R^3(t))c_\mathrm{p}}_{m_\mathrm{D}(t)} \qquad (14)$$

A derivation with respect to time using Leibniz integral rule yields Eq. (15) and (16), respectively.

$$0 = \frac{\delta}{\delta t}\left\{\int_0^{R(t)} 4\pi r^2 c_\mathrm{Cu}(r,t)dr + \frac{4}{3}\pi(R_0^3 - R^3(t))c_\mathrm{p}\right\} \qquad (15)$$

$$0 = \int_0^{R(t)}\frac{\delta c_\mathrm{Cu}(r,t)}{\delta t}\underbrace{4\pi r^2 dr}_{dv} + \int_0^{R(t)} c_\mathrm{Cu}(r,t)\underbrace{4\pi r^2\frac{dr}{dt}}_{\frac{dv}{dt}} - c_\mathrm{p}4\pi R^2(t)\frac{dR(t)}{dt} \qquad (16)$$

After replacing $\frac{\delta c_\mathrm{Cu}(r,t)}{\delta t}$ in Eq. (16) with the right-hand side of Eq. (11) and integration, Eq. (17) gives the moving boundary condition in Eq. (13) for $r = R(t)$.

$$0 = 4\pi R^2(t)D\frac{\delta c_\mathrm{Cu}(R(t),t)}{\delta r} + c_\mathrm{Cu}(R(t),t)4\pi R^2(t)\frac{dR(t)}{dt} - c_\mathrm{p}4\pi R^2(t)\frac{dR(t)}{dt} \qquad (17)$$

---

[3] The number of particles is required to convert the overall volume into a single particle volume and calculate changes in the particle radius.
[4] Note that $c_\mathrm{Cu2+}, c_\mathrm{CuO}$ and c_AA are molar concentrations (mmol/L), while the surface concentrations $c_\mathrm{Fe,S}$ and $c_\mathrm{Cu,S}$ are number concentrations (#/L).

A similar boundary condition was derived to model the swelling of polymeric particles in humid environments, using a fixed surface concentration[6]. Here, the copper surface concentration decreases with dissolution such that future surface concentrations depend on the subsequent radii and *vice versa*. With an explicit numerical scheme, the characteristic two-step dissolution behavior (observed in Figure 1b and Figure S2b,d,f) was obtained, but without conservation of mass (inherent issue of many explicit numerical schemes, especially for cases with large concentration gradients[7]). To ensure conservation of mass, the solution was split into the (1) fast release of surface available copper until the situation in Figure S5d with zero Cu at the surface is reached and (2) a diffusion limited solution, where the dissolution rate is given by the copper flux to the surface ($c_{\mathrm{Cu,s}}(t=0)=0$). The final release profile is superimposed of the two solutions (see Figure S5e).

The numerical solution for the spherical diffusion equation (PDE) coupled with the moving boundary condition (ODE) in Eq. (7) was obtained using a Landau transformation $z=\frac{r}{R(t)}$ $(0\leq z\leq 1)$ to fix the spartial dimension of the numerical grid[7-8]. With the total derivative $\frac{dc_{\mathrm{Cu}}(r,t)}{dt}=\frac{\delta c_{\mathrm{Cu}}(r,t)}{\delta r}\frac{\delta r}{\delta t}+\frac{\delta c_{\mathrm{Cu}}(r,t)}{\delta r}$ and a constant diffusion coefficient, Eq. (18) is the transformed partial DGL with the transformed boundary conditions Eq. (19) and (20).

$$\frac{dc_{\mathrm{Cu}}(z,t)}{dt}=\frac{z}{R(t)}\frac{dR(t)}{dt}\frac{\delta c_{\mathrm{Cu}}(z,t)}{\delta z}+\frac{D}{R(t)^2}\left[\frac{2}{z}\frac{\delta c_{\mathrm{Cu}}(z,t)}{\delta z}+\frac{\delta^2 c_{\mathrm{Cu}}(z,t)}{\delta z^2}\right] \tag{18}$$

$$\frac{dc_{\mathrm{Cu}}(0,t)}{dz}=0 \tag{19}$$

$$\frac{D}{R(t)}\frac{dc_{\mathrm{Cu}}(1,t)}{dz}=\left[c_{\mathrm{p}}-c_{\mathrm{Cu}}(R(t),t)\right]\frac{dR(t)}{dt} \tag{20}$$

To solve the moving boundary, Eq. (7) coupled with the concentration profiles given by Eq. (18), the method of lines (MOL) was used. The left-hand side of Eq. (18) was discretized using N nodes, resulting in the following ODE system.

$$\frac{dc_{\mathrm{Cu},i}}{dt}=\left[\frac{(i-1)\Delta z}{2R(t)}\frac{dR(t)}{dt}+\frac{D}{(i-1)\left(\Delta z R(t)\right)^2}\right]\left(c_{\mathrm{Cu},i+1}-c_{\mathrm{Cu},i-1}\right)+\frac{D}{\left(\Delta z R(t)\right)^2}\left(c_{\mathrm{Cu},i+1}-2c_{\mathrm{Cu},i}+c_{\mathrm{Cu},i-1}\right)\text{ for } i=2{:}\,N-1 \tag{21}$$

A discretization of the symmetry boundary condition in Eq. (19) was obtained using L'Hospital rule and the surface concentration was set to zero (Eq. (23)). The moving boundary condition becomes Eq. (24).

$$\frac{dc_{\mathrm{Cu},1}}{dt}=\frac{6D}{R(t)^2}\frac{c_{\mathrm{Cu},2}-c_{\mathrm{Cu},1}}{\Delta z^2} \tag{22}$$

$$\frac{dc_{\mathrm{Cu},N+1}}{dt}=0 \qquad (c_{\mathrm{Cu},N+1}=0) \tag{23}$$

$$\frac{dR(t)}{dt}=D\frac{M_{\mathrm{CuO}}}{\rho_{\mathrm{CuO}}N_A}\frac{3c_{\mathrm{Cu},N+1}-4c_{\mathrm{Cu},N}+c_{\mathrm{Cu},N-1}}{R(t)2\Delta z} \tag{24}$$

The DGL system for the complete model was solved in Matlab using ode15s ('RelTol' = 1e-10 and 'AbsTol' = 1e-12). Conservation of mass was validated for the complete removal of copper from the particle, *i.e.* by comparison of the simulated particle diameter for the case when all copper is released with the theoretically expected particle diameter for each initial iron-copper ratio, as shown in Table S5. All fit parameter in Table S1 were determined using fmincon in Matlab. The kinetics were validated in simplified model media to demonstrate the transformation on the nanoparticle/medicine side. In more complex cellular environments, factors such as physisorption of polypeptides[9], protein corona formation or agglomeration behavior may need to be taken into account[10]. Even though excluded in the model, agglomeration may play an important role for the copper bioavailability under high particle concentrations used in cancer treatments. With an increasing iron-copper ratio during the dissolution process this effect enhances at neutral pH, because the isoelectric point of Fe_3O_4 is 6.5[11], while of CuO about 9.5[11]. Less agglomeration under more acidic conditions in certain cancer cell types could possibly even increase the therapeutic window. However, the presence of certain species such as sodium bicarbonate with the possible formation of ferrous bicarbonate, causes a complete dissolution of Fe-doped CuO NPs. Hence, a release rate constant k_{Fe} needs to be considered in respective media.

XRD, TEM and EDX measurements. X-ray powder-diffraction data for all samples were collected on a Bruker D8 Discover equipped with a θ-θ Bragg-Brentano-Goniometer, Cu-$K_{\alpha1}$,$K_{\alpha2}$ radiation monochromatized by high-resolution energy discrimination in a LynxEye XE-T linear position sensitive detector with 192 channels, with apertures of 3.296°2θ in total and 0.017167°2θ/channel. The samples were prepared as powders dispersed in a thin layer on a flat, nominally background-free single-crystal Si disk and rotated about the disk-normal with 4s/rotation. Data were collected from 5 – 138°2θ in steps of 0.01488°2θ with an integral counting time of 288s/step. Single-crystal X-ray diffraction data of Cu(II)-L-glutamine were collected with a Bruker D8 Venture diffractometer using MoKα radiation (λ = 0.71076 Å), equipped with curved TRIUMPH monochromator, 0.3 mm collimator, four-circle κ-goniometer and Photon 100 CMOS area detector. Data collection parameters and crystal data are listed in Table S3. For TEM measurements, a small portion of CuO-type NPs (~1-2 mg) was dispersed in 5mL of ethanol (AR grade, Strem) in an ultrasonic bath and sonicated for 15 minutes. A drop of the sample solution was placed on a carbon-coated copper grid. The samples were dried at ambient conditions and loaded in a FEI Titan

80/300 microscope equipped with a Cs corrector for the objective lens, a Fischione high angle annular dark field detector (HAADF), GATAN post-column imaging filter and a cold field emission gun operated at 300 kV acceleration voltage. The chemical composition of the samples prior and post dissolution was analysed with scanning electron microscope Leo 1530 (Gemini), equipped with an EDX detector (X-Flash 6/30, Bruker with a 30 mm² detector area). The samples were Au-sputtered (40 s at 20 mA, K550, EMITECH) to avoid charging during the measurement.

Electron paramagnetic resonance spectroscopy. CuO NPs were suspended in milli-Q H_2O at a concentration of 200 ppm. The suspensions were sonicated for 20 min and used as stock solutions for further dispersion in <RPMI+10% fetal bovine serum (FBS) medium> at a concentration 50 ppm (as in[1]) and higher particle concentrations. Electron Paramagnetic Resonance (EPR) spectra were recorded with a Bruker ER200D spectrometer at liquid nitrogen (77 K) temperatures equipped with an Agilent 5310 A frequency counter.

Cell cultures. Cell types used: murine mesenchymal stem cells (MSC), human bronchial epithelial cells (Beas-2B), murine lung squamous tumor cells (KLN 205) and human cervical cancer cells (HeLa). Beas-2B and HeLa cells were cultured in high glucose containing Dulbecco's modified Eagle's medium (DMEM), supplemented with 10% fetal calf serum, 1 mM sodium pyruvate, 2 mM L-glutamine, and 1% penicillin/streptomycin (Gibco, Invitrogen, Belgium). MSCs were cultured in high glucose DMEM, supplemented with 10% fetal calf serum, 10% horse serum, 1 mM sodium pyruvate, 2 mM L-glutamine, and 1% penicillin/streptomycin. KLN 205 cells were cultured in high glucose DMEM, supplemented with 10% fetal calf serum, 1 mM sodium pyruvate, 2 mM L-glutamine, 1% non-essential amino acids and 1% penicillin/streptomycin. All cell types were maintained in a humidified atmosphere at 37°C and 5% CO_2 and split 1/5 upon reaching 80% confluency.

Cell-nanoparticle interaction studies. For high-content imaging studies, all cell types were seeded at 3000 cells/well in a 96 well plate (Nunc, Belgium) after which the cells were allowed to attach overnight in a humidified atmosphere at 37°C and 5% CO_2. Subsequently, the cells were incubated with the different CuO NPs for 8 h in their full growth medium at concentrations of 0, 5, 10, 15, 20, 25, 30 and 35 µg/ml. Every condition was performed in triplicate and results were analyzed based on the three repeats. The high-content imaging experiments were performed based on previously validated methods. Experimental details are given in the following sections.

Generation of Doxorubicin-resistant cancer cells. To test the effectivity of CuO NPs against drug-resistant cancer cells, doxorubicin-resistant HeLa and KLN 205 cells were generated. The cells were seeded in 25 cm² flask at 100,000 cells/flask after which they were allowed to settle overnight. Next day, the cells were given fresh medium, containing 500 pM doxorubicin (Sigma Aldrich) and were kept in culture for three days. After three days, cells were passaged and reseeded in 25 cm² flasks at 100,000 cells per flask, where they were grown in medium containing 1.5 nM doxorubicin. Cells were then passaged when reaching 80% confluency, after which they were reseeded in 25 cm² flasks at 100,000 cells per flask, where they were now grown in medium containing 4.5 nM doxorubicin. This process was repeated four times, using doxorubicin concentrations of 12.5 nM, 37.5 nM, 112.5 nM and 337.5 nM, respectively. The last values were well above the IC50 values reported for HeLa (143 nM[12]) and KLN 205 (203 nM, empirically determined). Cells were then kept at 337.5 nM doxorubicin and labeled as KLN 205-R or HeLa-R cells.

Cell viability in co-culture experiments. For co-culture experiments, all cell types were seeded in 25 cm² flasks at 200,000 cells/flask and allowed to settle overnight in a humidified atmosphere at 37°C and 5% CO^2. Next day, the cancer cells (KLN 205, HeLa, KLN 205-R or HeLa-R) were labeled with CellTracker Red CMTPX (4 µM, for 30 min) in full media at 37°C. After labelling, cells were washed twice with PBS, trypsinized and reseeded at 750 cells/well in a 96 well plate (Nunc, Belgium). Non-labeled normal cells (MSC, Beas-2B) were also reseeded into the same wells at 750 cells/well. Overall, we made 8 combinations of different cell cultures: MSC + red KLN 205(-R), MSC + red HeLa(-R), Beas-2B + red KLN 205(-R), Beas-2B + red HeLa(-R). The media composition of the co-cultures consisted of 50 µl of medium 1 (normal cell types) and 50 µl of medium 2 (cancer cell types). All cells were allowed to attach overnight in a humidified atmosphere at 37°C and 5% CO^2. Then, the cells were incubated with the different 6% Fe-doped CuO NPs for 24 h in the full growth medium at a concentration of 12.5 µg/ml. Every condition was performed in triplicate and results were analyzed based on the three repeats. Following cellular exposure to the NPs, cells were washed twice with phosphate buffered saline (PBS; Gibco, Invitrogen, Belgium) and treated with 2 µM fixable Live-Dead Green dead cell stain (Molecular Probes, Life Technologies Europe, BV, Belgium) in 100 µl/well of PBS (with Ca^{2+} and $Mg^{2+)}$ and incubated in the dark for 30 min at room temperature. Next, the staining media was aspirated, cells were washed gently with PBS (3x) fixed with 4% paraformaldehyde (PFA) for 15 min at room temperature. The fixative was aspirated and cells were washed three times with PBS. Cells were then counterstained using Hoechst 33342 Nuclear stain (20 µg/ml PBS in 100 µl/well) for 15 min at ambient temperature in the dark. The nuclear counterstain was then removed, cells were washed three times with PBS and 100 µl of PBS was added to every well, after which the plates were analyzed using the InCell 2000 analyzer (GE Healthcare Life Sciences, Belgium). During acquisition, a minimum of 5000 cells/condition were acquired (over 3 wells) using a 20x objective for the following channels: UV/blue for Hoechst nuclear stain, FITC/FITC for the Live-Dead Green dead cell stain and TexasRed/TexasRed for the CellTracker Red CMPTX stain. Data analysis was performed analogous to the previous sections. The level of cell viability was calculated as follows: First, cells were segmented based on the Hoechst stain and the perinuclear region was determined by enlarging the nuclear stain 2.5-fold and using the original Hoechst stain images as seed images. The TexasRed/TexasRed channel was then segmented and overlapping red and enlarged blue stains were defined as the cancer cells. Then, the FITC/FITC channel was segmented (green stain for dead cells) and overlapping green and blue stains were defined as dead

cells, overlapping green, blue and red stains were defined as dead cancer cells. Cell viability was then calculated by determining the number of total cells minus the number of dead cells (dead cells are defined as cells with clear green nuclei, where the intensity is minimally 3-fold above noise level and having an area of minimally 2 µm²). These values were then normalized to control values (100%) to indicate the degree of cell viability.

Mice experiments. Female DBA/2 mice (Harlan Laboratories, Cambridgeshire, UK), 5–7 weeks old, were used in this study. The animal studies used a syngeneic tumor model in which DBA/2 animals received 500,000 KLN 205 cells or KLN 205-R cells in 200 µl saline as a subcutaneous injection on the lower part of the left side of the back. For detailed follow-up studies, Balb/c mice bearing syngeneic CT26 colon carcinoma following subcutaneous engraftment in the lower part of the back were used. All mouse surgical procedures and imaging were performed with the animals anesthetized by inhalation of 2% isoflurane. The condition of the animals was monitored every day and their weight was measured every other day. Tumors were measured with calipers every other day. When tumors reached the size of minimally 50 mm³ (approximately 10-14 days after tumor inoculation), the animals were divided into different groups of similar tumor size for further experiments. When tumors became larger than 1.5 cm or a deep ulcer was formed, euthanasia was performed. If animal weight dropped by 10%, the animals were sacrificed. For the tumor growth studies, all animals were sacrificed 5 weeks after surgery NP administration. For euthanasia, animals were subjected to 5% isoflurane inhalation. To ensure death following isoflurane inhalation cervical dislocation was performed. Tumors were then removed from the animals and used for *ex vivo* imaging. All animal studies were approved by KU Leuven's Institutional Animal Care and Use Committee (IACUC; approval number P259/2015) in accordance with the principals and procedures outlined in national and European regulations.

Monitoring of tumor growth. DBA/2 mice with fLuc-expressing KLN 205 tumors of minimally 50 mm³ were divided into different groups containing 4 animals per group. Before each imaging session, the mice were injected intraperitoneally with 126 mg/kg D-luciferin (Promega, Madison, WI, USA) dissolved in PBS (15 mg/ml). Next, all 4 animals per group were positioned in the IVIS Spectrum and images were acquired after 10 min under 2% isoflurane inhalation. The animals received a peritumoral injection of either 100 µl of saline (control animals) or 100 µl saline containing CuO (pure CuO, 6% or 10% Fe-doped CuO NPs) at 125 µg/animal. In later repeat studies, animals received higher doses of 6% Fe-doped CuO NPs at 175 or 225 µg/animal. As controls, both wild-type and resistant tumor-bearing animals were also treated with Doxorubicin at two different doses, being either 2 or 4 µmol/kg bodyweight. In additional follow-up groups, animals received 100 mg/kg/day epacadostat (INCB024360, SeleckChem) by oral gavage, starting at the same time of initial NP treatment. For repeated studies, animals received a second bolus of NPs 14 days following the initial administration. Images were acquired just before NP or saline injection and after 5, 10, 15, 21, 28 and 35 days post NP injection (medium binning, f stop = 1, time = 25 seconds). Bioluminescence images were analyzed using the LivingImage (Perkin Elmer, Waltham, MA) processing software. Regions of interest (ROIs) were drawn around the bioluminescent signals in the tumor regions of the mice, and measurements were generated as the total flux (p/s/cm^2/sr) from the selected ROIs.

Statistical analysis. Statistical analysis of cell and mice experiments was performed using GraphPad Prism 6.0, where all data were analyzed using one-way ANOVA. For statistical significance of any treated group from the control group or between two treated groups under one condition, a Dunnett's post-hoc test was applied.

High content cell analysis.
Cell viability, membrane damage and mitochondria. Following cellular exposure to (Fe-doped) CuO NPs, cells were washed twice with phosphate buffered saline (PBS; Gibco, Invitrogen, Belgium) and treated with 200 nM MitoTracker Red CMXRos and 2 µM fixable Live-Dead Green dead cell stain (Molecular Probes, Life Technologies Europe, BV, Belgium) in 100 µl/well of PBS (with Ca^{2+} and Mg^{2+}) and incubated in the dark for 30 min at room temperature. Next, the staining media was aspirated, cells were washed gently with PBS (3x) fixed with 4% paraformaldehyde (PFA) for 15 min at room temperature. The fixative was aspirated and cells were washed three times with PBS. Cells were then counterstained using Hoechst 33342 Nuclear stain (20 µg/ml PBS in 100 µl/well) for 15 min at ambient temperature in the dark. The nuclear counterstain was then removed, cells were washed three times with PBS and 100 µl of PBS was added to every well, after which the plates were analyzed using the InCell 2000 analyzer (GE Healthcare Life Sciences, Belgium). During acquisition, a minimum of 5000 cells/condition were acquired (over 3 wells) using a 20x objective for the following channels: UV/blue for Hoechst nuclear stain, FITC/FITC for the Live-Dead Green dead cell stain and DsRed/DsRed for the MitoTracker Red CMXRos stain. Data analysis was then performed on the InCell Investigator software (GE Healthcare Life Sciences, Belgium) using in-house developed protocols. The level of cell viability was calculated as follows: First, cells were segmented based on the Hoechst stain and the perinuclear region was determined by enlarging the nuclear stain 2.5-fold and using the original Hoechst stain images as seed images. Cell viability was calculated by determining the number of total cells minus the number of dead cells (dead cells are defined as cells with clear green nuclei, where the intensity is minimally 3-fold above noise level and an area of minimally 2 µm²). These values were then normalized to control values (100%).

For membrane damage, the analysis occurred similarly. All green dots in the perinuclear area with a minimum intensity of 3-fold above the noise level and with a size of minimally 0.1 µm² but smaller than 2 µm² were selected. The ratio of this value with the value obtained for control cells was then given to indicate the level of membrane damage.

The mitochondrial stress and ROS were calculated as follows: the DsRed/DsRed channel was segmented, using the nuclear target channel as seed images. Based on the segmented mitochondrial images, the overall area of cellular mitochondria were calculated, for

any dot in the mitochondrial channel that had an intensity of minimum 3-fold higher than the background noise level. The total area of cellular mitochondria was determined as a marker for mitochondrial stress, where damaged mitochondria change shape, turning from an elongated to a more spherical morphology. The total area of cellular mitochondria was then normalized to the area of mitochondria in untreated control cells (100%). For mitochondrial ROS, the level of fluorescence intensity of the segmented mitochondria was determined. The intensity of the mitochondrial signal was then normalized to the intensity level of untreated control cells (100%).

Cell morphology. After cellular exposure to (Fe-doped) CuO NPs, cells were washed (3x) with 500 µl PBS/well and fixed for 15 min at room temperature with 4% PFA. The fixative was then aspirated, cells were washed (3x) with PBS (500 µl/well) after which cells were permeabilised with 250 µl/well of Triton X-100 (1%) for 10 min at room temperature. Cells were then blocked with 10% serum-containing PBS for 30 min at room temperature. Next, cells were stained using 100 µl of staining solution per well of Acti-Stain 488 (Tebu-Bio, Belgium) and incubated for 90 min in the dark at room temperature. The staining solution was aspirated, cells were washed (3x) with PBS (100 µl/well) after which 100 µl fresh PBS was added to each well and the plates were kept at 4°C in a dark container until analyzed using the InCell 2000 high-content imaging system. For acquisition, the following channels were selected: UV/blue for Hoechst nuclear stain, and FITC/FITC for the actin stain. Data analysis was then performed on the InCell Investigator software (GE Healthcare Life Sciences, Belgium) using in-house developed protocols, using a minimum of 5000 cells/condition. The size of the cells was calculated as follows: First, cell nuclei were segmented based on the blue channel. Cells were then segmented using the FITC channel, where any holes in the cells were filled up and included. Cells on the border of the field of view were excluded from the analysis. The segmentation was based on the blue channel as seed channel for the nucleus. The total area of every individual cell was then determined.

Autophagy. After cellular exposure to the (Fe-doped) CuO NPs, cells were washed (3x) with 100 µl PBS/well and fixed for 15 min at room temperature with 4% PFA. The fixative was then aspirated, cells were washed (3x) with PBS (100 µl/well) after which cells were permeabilised with 50 µl/well of Triton X-100 (1%) for 10 min at room temperature. Cells were then blocked with 10% serum-containing PBS (blocking buffer) for 30 min at room temperature. Next, cells were stained using 100 µl of staining solution per well consisting out of primary mouse anti-LC3 antibody (1/400 dilution in blocking buffer; Cell Signalling Technologies, Belgium) and incubated for 90 min in the dark at room temperature. The primary antibody solution was aspirated, cells were washed (3x) with blocking buffer (100 µl/well) after which 75 µl of secondary AF488-conjugated goat anti-mouse IgG antibody (1/250 dilution in blocking buffer; Molecular Probes, Belgium) was added to each well and plates were incubated in the dark for 60 min at room temperature. Following this, the incubation media was aspirated, cells were washed (3x) with PBS (100 µl/well), after which 100 µl fresh PBS was added to each well and the plates were kept at 4°C in a dark container until analyzed using the InCell 2000 high-content imaging system. For acquisition, the following channels were selected: UV/blue for Hoechst nuclear stain, and FITC/FITC for the LC3 stain. Data analysis was then performed on the InCell Investigator software (GE Healthcare Life Sciences, Belgium) using in-house developed protocols, using a minimum of 5000 cells/condition.

The level of autophagy was calculated as follows: First, cell nuclei were segmented based on the blue channel. Using the FITC channel, the cell cytoplasm was then selected, and cells were segmented, where any holes in the cells were filled up and included and any cells on the border of the field of view were excluded from the analysis. The segmentation was based on the blue channel as seed channel for the nucleus. Using the original FITC/FITC channel, any green dots having an intensity of minimum twice that of the noise level and that were localized within the cytoplasm were segmented, where multiple green dots could be localized within a single cell cytoplasm. Then, the cellular intensity of the green channel was measured for every cell, after which this value was normalized to the control value (100%).

ImageStream analysis. GFP-CT26 tumor-bearing Balb/c mice were either left untreated, treated with epacadostat or 6% Fe-doped CuO + epacadostat and treatment was complete, animals were sacrificed (4 animals per group) and tumors were isolated, treated with collagenase and DNase, followed by treatment with a MACS Tissue Dissociator followed by straining the cell suspensions through a tissue strainer. Cells were then sorted to remove GFP positive tumor cells (FacsAria Cell Sorter) and the remaining cells were fixed with 2% PFA for 20 min at room temperature and split into 2 different vials (500 µL each), washed with PBS containing 5% FBS (Sigma Aldrich) and Fc receptors were blocked using murine FcR blocking reagent (Miltenyi). Cells were then stained in staining buffer (1% bovine serum albumin in PBS) with fluorescently tagged antibodies. For group 1, antibodies were: eFluor405-anti F4/80 (macrophage marker; 3 µg/ml), FITC-anti-CD3 (T cell marker, 3 µg/ml), PE-anti-NK1.1 (NK cell marker, 3 µg/ml), APC-anti-CD19 (B cell marker, 1 µg/ml); for group 2, antibodies were: FITC-anti-CD4 (CD4 T cells, 3 µg/ml), PE-anti-CD8 (CD8 T cells, 3 µg/ml), APC-anti-CD69 (T cell activation, 3 µg/ml) for 30 minutes, followed by centrifugation, washing the cells and resuspending the cells in 50 µl PBS followed by image-based flow cytometry analysis. Data was acquired using an ImageStream X MkII (ISX, Amnis, Seattle, USA) equipped with 405, 488, 561 and 642 nm excitation lasers. All samples were acquired at ×40 magnification with a 0.7 numerical aperture (NA) objective. A minimum of 10000 cells were collected for each sample. Only data from relevant channels were collected including. Data from samples with only single stains were also captured to calculate the compensation matrix required to account for spectral overlap between the chosen fluorophores. Image analysis was performed using the IDEAS software (Amnis, Seattle, USA), allowing selection of single, focused cells, after which gating was performed based on analyzing the different fluorescent channels. The same analysis protocol was applied for every sample of each group, enabling a precise comparison of different samples (treated *versus* untreated).

Blood biochemistry analysis. DBA/2 mice with fLuc-expressing KLN 205 tumors of minimally 50 mm³ were divided into different groups containing 4 animals per group. Animals were treated with either saline, epacadostat, 6% Fe-doped CuO (225 µg/mouse) or 6% Fe-doped CuO (225 µg/mouse) + epacadostat. Blood samples were collected retroorbitally following animal sacrifice (200 µl/animal), and samples were collected and centrifuged in heparin-containing tubes to separate plasma from serum (15 min at 3500 rpm). Next, 75 µl serum was added on analysis discs (Samsung Comprehensive test 16V) enabling analysis of 16 different markers using the Samsung PT10V chemistry analyzer (SCIL Animal care company GmbH, Viernheim, Germany). The following markers were analyzed: glucose, urea, creatinine, urea/creatinine ratio, phosphates, calcium, total protein, albumin, globulin, albumin-globulin ratio, alanine aminotransferase, alkaline phosphatases, bilirubin, cholesterol, triglycerides and amylase.

Monitoring of neutrophil influx. CT26-bearing Balb/c mice with tumors of minimally 50 mm³ were divided into different groups containing 4 animals per group. Animals were treated with either saline, doxorubicin (5 µmol/kg) + epacadostat or 6% Fe-doped CuO (225 µg/animal) + epacadostat. On day 1 and day 6 post treatment, animals were injected with 4 nmol Neutrophil Elastase 680 FAST (Perkin Elmer) by tail vein injection, and animals were scanned in the IVIS Spectrum after 4 hours. Images were acquired using 20 sec exposure time, (medium binning, f stop = 1, Ex: 675 nm, Em: 720 nm). Fluorescence images were analyzed using the LivingImage (Perkin Elmer, Waltham, MA) processing software. Regions of interest (ROIs) were drawn around the fluorescent signals in the tumor regions of the mice, and measurements were generated as the total flux (p/s/cm^2/sr) from the selected ROIs.

Monitoring of *in-vivo* caspase activity. CT26-bearing Balb/c mice with tumors of minimally 50 mm³ were divided into different groups containing 4 animals per group. Animals were treated with either saline, doxorubicin (5 µmol/kg) + epacadostat or 6% Fe-doped CuO (225 µg/animal) + epacadostat. On day 1 and day 6 post treatment, animals were injected with 4 nmol fluorescent *in vivo* pan caspase probe (655-VAD-FMK) (Vergent Bioscience) by tail vein injection, and animals were scanned in the IVIS Spectrum after 4 hours. Images were acquired using 20 sec exposure time, (medium binning, f stop = 1, Ex: 625 nm, Em: 680 nm). Fluorescence images were analyzed using the LivingImage (Perkin Elmer, Waltham, MA) processing software. Regions of interest (ROIs) were drawn around the fluorescent signals in the tumor regions of the mice, and measurements were generated as the total flux (p/s/cm^2/sr) from the selected ROIs.

Tissue immunofluorescence. CT26-bearing Balb/c mice with tumors of minimally 50 mm³ were divided into different groups containing 4 animals per group. Animals were treated with either saline, doxorubicin (5 µmol/kg) + epacadostat or 6% Fe-doped CuO (225 µg/animal) + epacadostat. At 10 days following treatment, animals were sacrificed, tumors were removed, fixed in 2% PFA for 24 h and snap-frozen in OCT. Tissues were then processed with a cryotome into 10 µm thick sections and placed on histological slides. Slides were then heat-treated for antigen retrieval, blocked with 10% serum-containing medium, and stained with primary goat-anti-mouse F4/80 antibody (2 µg/ml, Abcam) and rabbit-anti-mouse CD8 antibody (2 µg/ml, Abcam) overnight at 4°C. Tissue sections were then washed in PBS three times and stained with secondary antibodies (AF488 donkey anti-goat and Cy3 donkey anti-rabbit; 4 µg/ml, Thermo Fisher Life Sciences) for 4 hrs. Tissues were then washed with PBS, counterstained with Hoechst and mounted with coverslips using Wako mounting medium. Slides were then visualized using a Zeiss Axioscan Slide Scanner Z.1 at 20x magnification and images were automatically stitched together and generated as a whole image per tissue section.

Results and Discussion

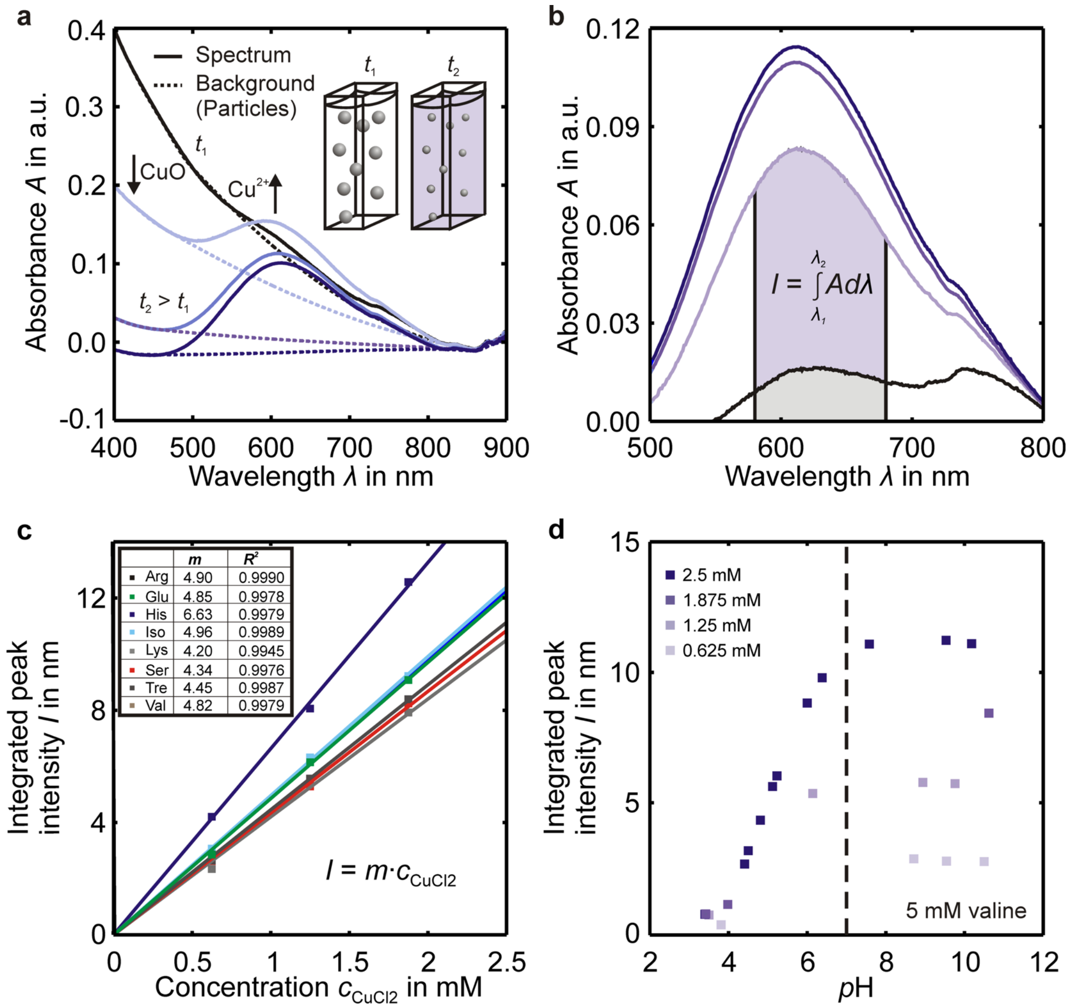

Figure S1. Pharmacokinetic measurements *via* UV-Vis. (a) Time dependent dissolution UV-Vis spectra of 2.5 mM CuO in 5 mM valine solution. Spectra are composed of (1) absorbance during band gap excitation and (2) absorbance due to Cu^{2+}-amino acid complexes with maxima centered at ~600 nm. While the signal intensity decreases for the particles, the signals for Cu^{2+}-amino acid increases with time. (b) To extract the Cu^{2+} signals (centered at ~600 nm) from the overlapping background including the NPs contribution, a background subtraction (dashed lines in Figure S1a) was conducted. Subsequently, the corrected spectra were integrated in the region between 580 to 680 nm to obtain signal intensities ***I*** proportional to the Cu^{2+} concentrations. (c) To convert the integrated signal intensities ***I*** to Cu^{2+}-concentrations, a calibration was done for the investigated amino acid solutions using $CuCl_2$. The pH values of the $CuCl_2$ solutions were buffered with 44 mM $NaHCO_3$. The CuO and amino acid concentrations for the dissolution profiles were chosen such that the peak intensities were clearly traceable within the linear region of absorbance < 0.5. (d) At pH > 7, the intensities were pH independent, while in acidic in environments the intensities depend on pH, which needs to be considered.

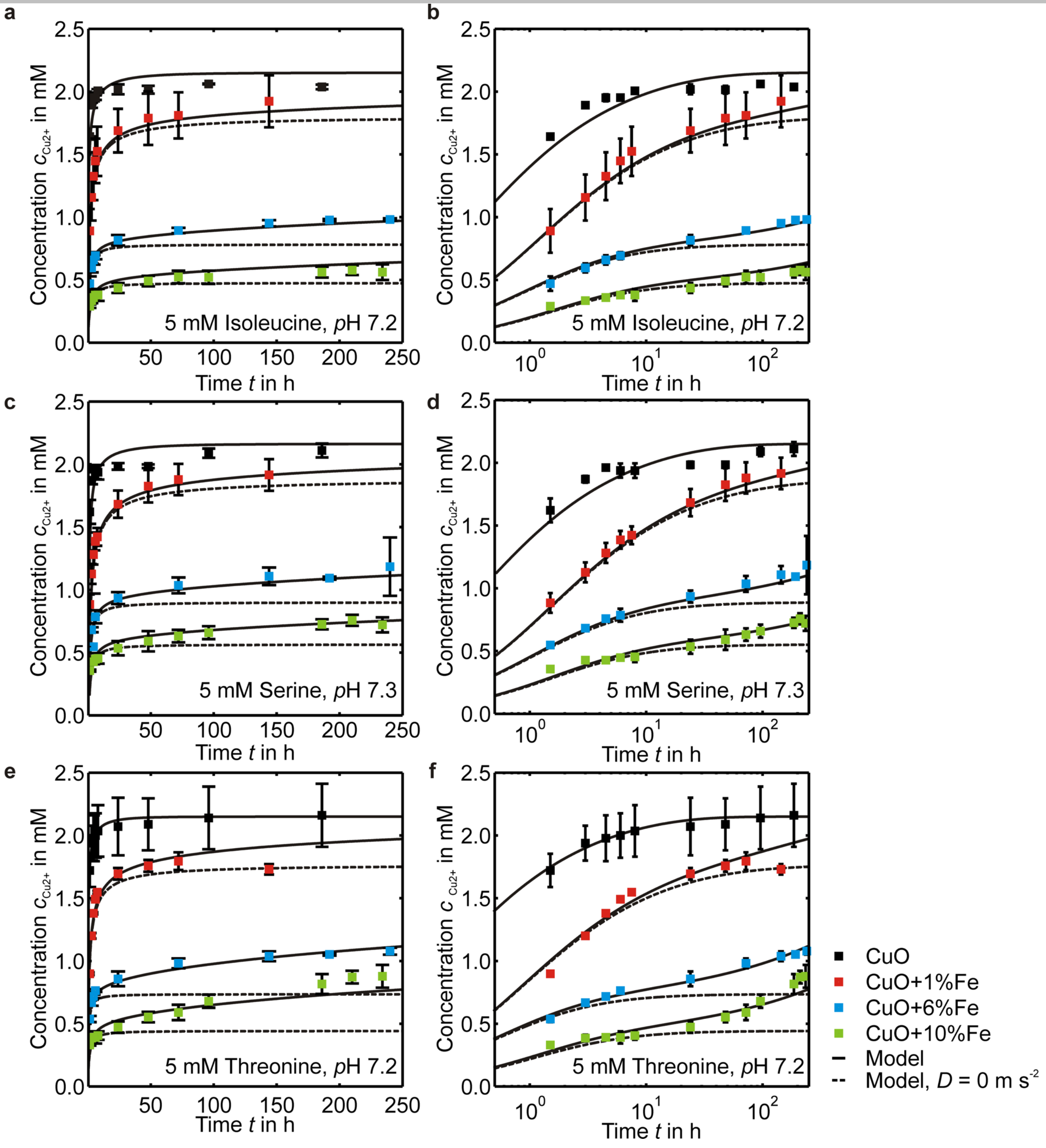

Figure S2. Pharmacokinetics for pure and Fe-doped CuO NPs. (a-f), Cu^{2+} release profiles for pure, 1, 6 and 10% Fe-doped CuO NPs in 5 mM isoleucine, serine and threonine solutions, respectively. The same two-step dissolution behavior, as described in the manuscript for valine, was observed for 6 and 10% Fe-doped samples on the logarithmic time scale. All fit parameter are given in Table S1 and explained in the main text and experimental procedures (Kinetic model).

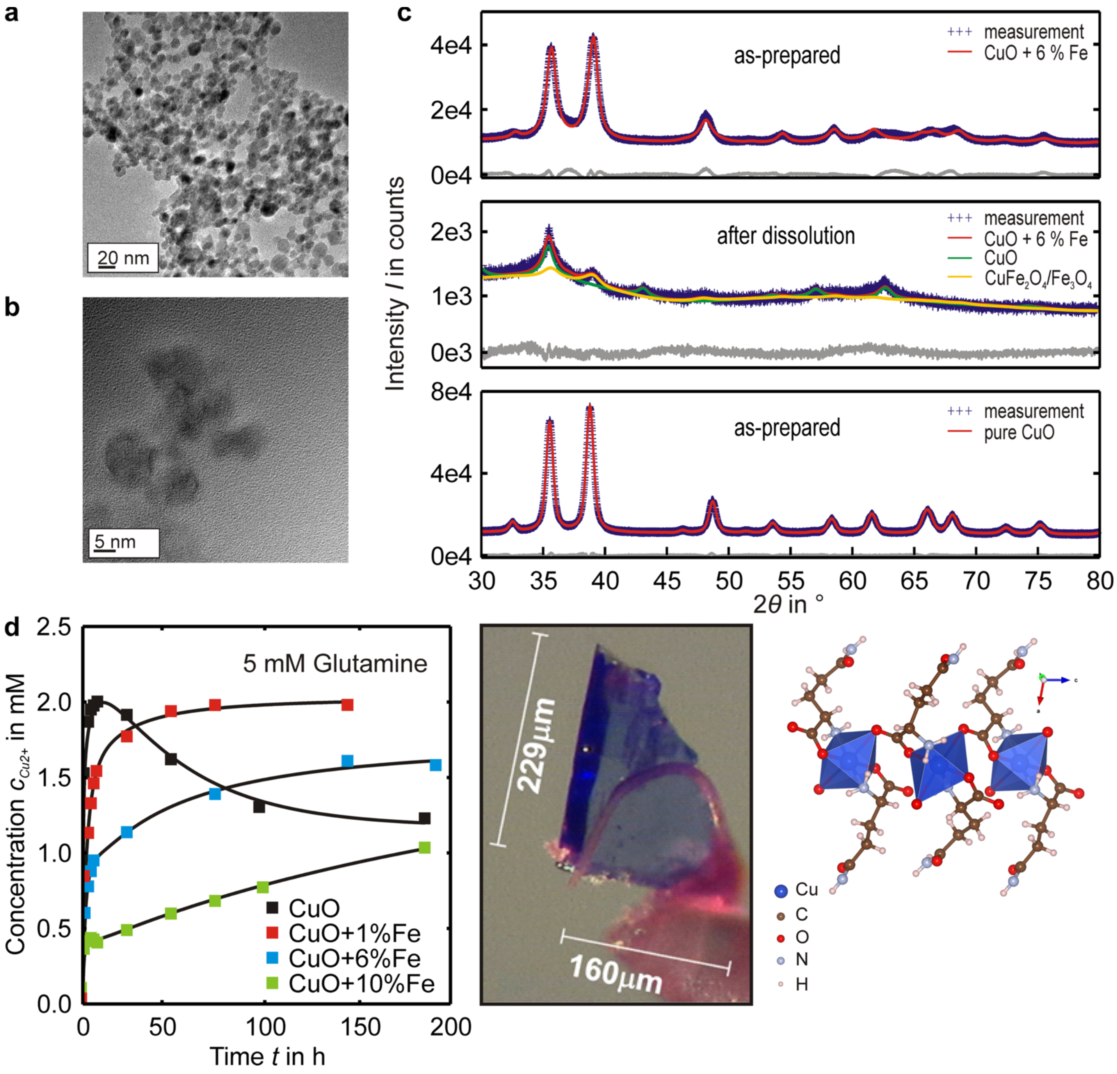

Figure S3. Particle characterization prior and post dissolution. (a,b) 6% Fe-doped CuO NPs as-prepared and after dissolution for four weeks in 50 mM valine solution (replaced in regular intervals to sustain the dissolution of a sufficient amount of particles for powder diffraction). Compared to the 10% Fe-doped NPs (Figure 1 c,d), the particle size decreased even more and less spherical particles remained after dissolution. (c) Powder diffraction (XRD) of 6% Fe-doped samples, before and after dissolution and a reference pattern of pure CuO NPs (as-prepared). (d) Dissolution behavior of CuO in 5 mM glutamine solution showed a decreasing Cu^{2+} signal after an initial increase (fast initial dissolution). This decrease is caused by complexation followed by crystallization/precipitation of Cu^{2+}-amino acid. The crystal structure of the light-blue single-crystals forming the precipitates was obtained from SC-XRD analysis evidencing the Cu^{2+} complexation in amino acid solution.

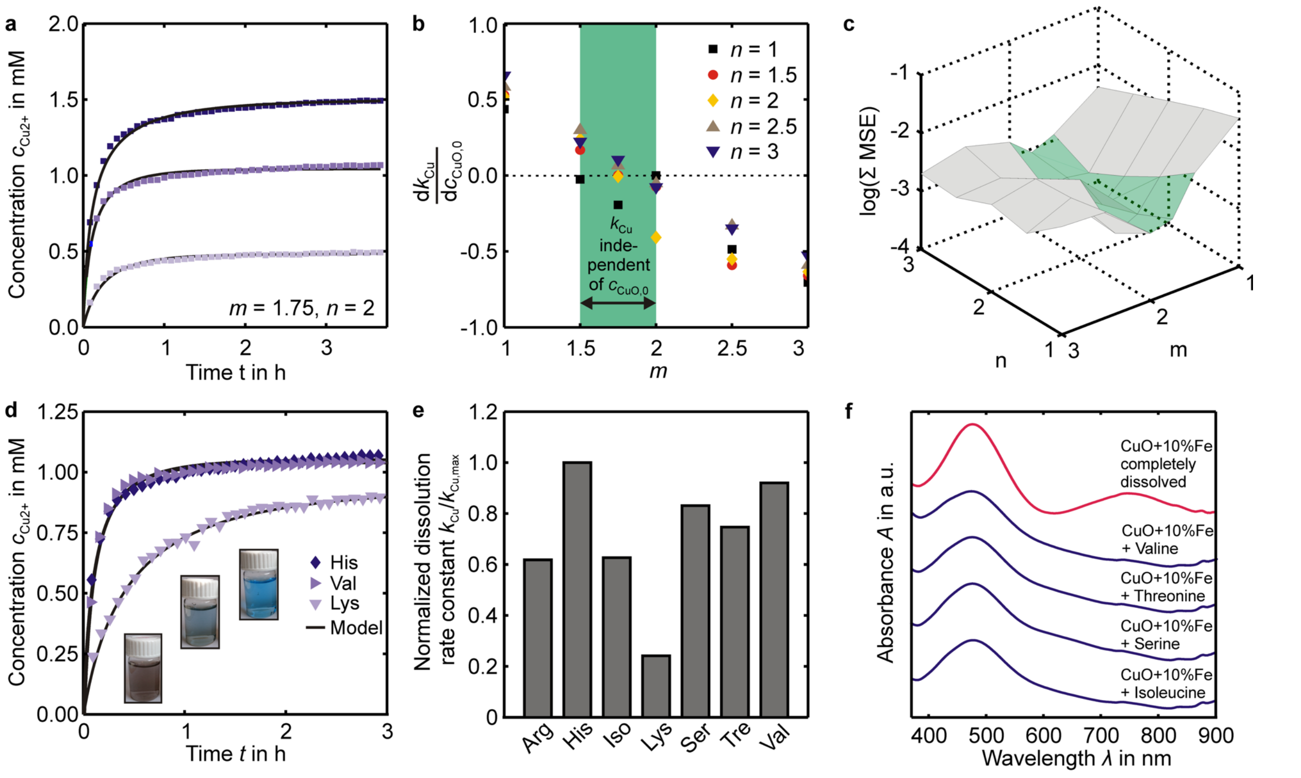

Figure S4. Pharmacokinetic measurements and model validation. (a) Experimental and modelled CuO dissolution kinetics (5 mM valine solution) with partial reaction orders $m = 1.75$ and $n = 2$. (b) Gradient in the dissolution rate constant with the initial CuO concentration $dk_{Cu}/dc_{CuO,0}$ as a function of the partial reaction order m. The dissolution rate constant k_{Cu} is independent of the concentration (zero gradient). This is the case for m in the range of 1.5-2. (c) Commulative mean square error between experiment and model as a function of the partial reaction orders m and n. In the green region, where k_{Cu} is independent of the concentration, a minimum mean square error was observed for n = 2 indicating a stoichiometric case (two amino acids complex with one Cu^{2+} ion, see SC-XRD in Figure S3d). (d) Dissolution profiles of 1.25 mM CuO in various 5 mM amino acid solutions. For 5 mM histidine and valine solutions the particles completely dissolved after 1 hour, while the dissolution proceeds much slower in 5 mM lysine solution. (e) Dissolution rates determined through fitting the model to experimental data shows a binding selectivity towards the amino acids. (f) A spot test for Fe^{3+} in solution was carried out for Fe-doped CuO NPs after long-term dissolution using potassium hexacyanoferrate(III). Completely dissolved particles at pH 2.5 give a positive control spectrum, while a release of Fe from the particles in amino acid solutions was undetectable at physiological pH.

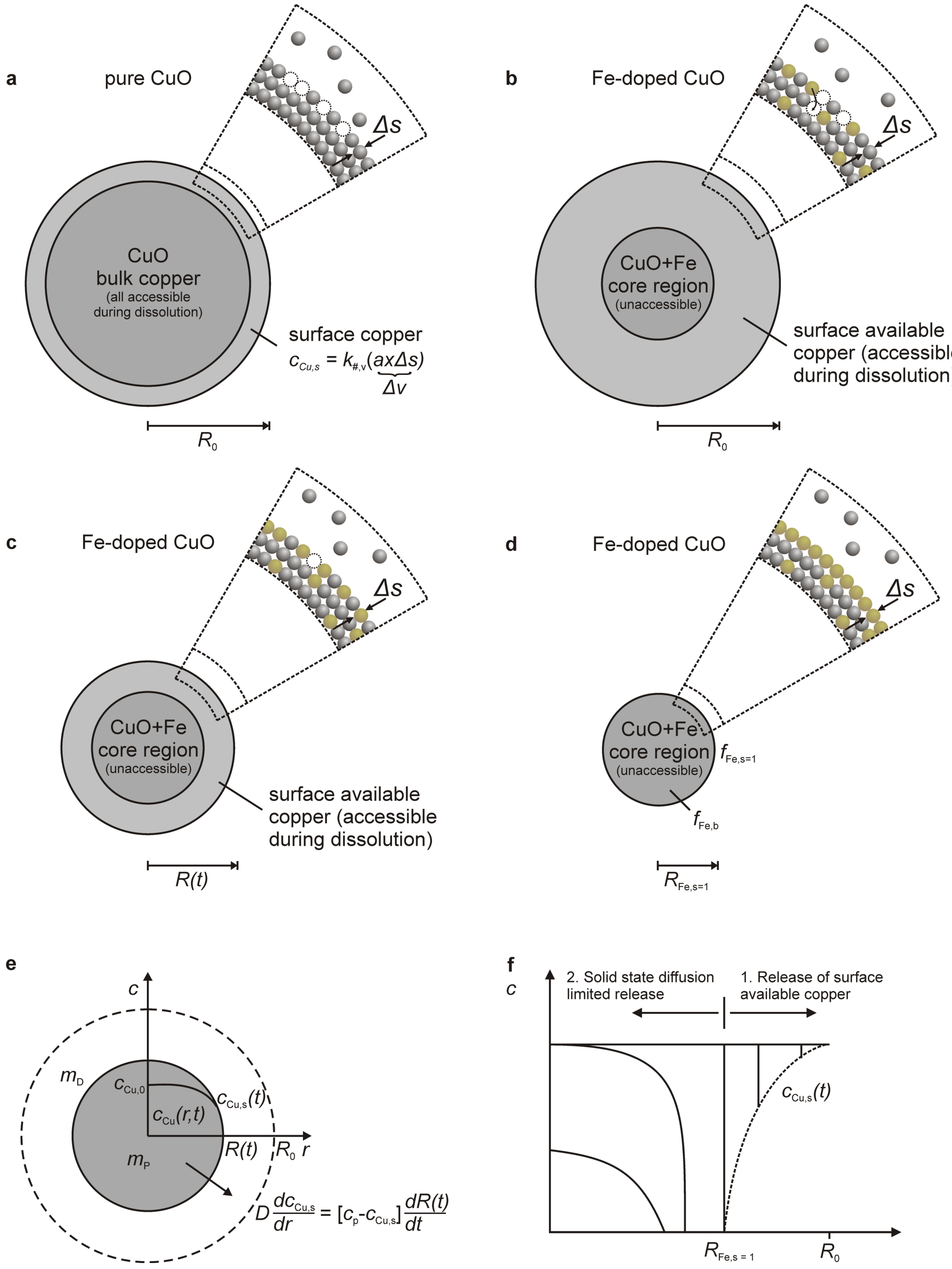

Figure S5. Visualization of model assumptions for the two-step dissolution of Fe-doped CuO NPs. (a) Cu^{2+} release from pure CuO NPs is proportional to the surface copper concentration $c_{\mathbf{Cu,S}}$ on the shrinking surface area. During dissolution all copper becomes accessible. (b-d), For Fe-doped CuO, the surface iron-copper ratio increases during dissolution until all surface available copper is released, *i.e.* only Fe remains in the surface shell. The dissolution stops at this point, if solid state diffusion from the CuO+Fe core region is excluded. The amount the surface available copper depends on the initial iron-copper ratio $f_{\mathbf{Fe}}$ and the particle size $d_{\mathbf{p,0}}$. In this schematic, the presence and release of lattice oxygen to form reactive oxygen species was omitted to demonstrate the model concept. (e) The moving boundary condition for the spherical diffusion equations was derived from a mass balance at the dissolving particle. (f)To obtain a numerical solution obeying conservation of mass, the situation in Figure S5e was split into (1) the fast release of surface available copper and (2) the solid state diffusion limited release assuming a surface copper concentration of zero. Model details in experimental procedures.

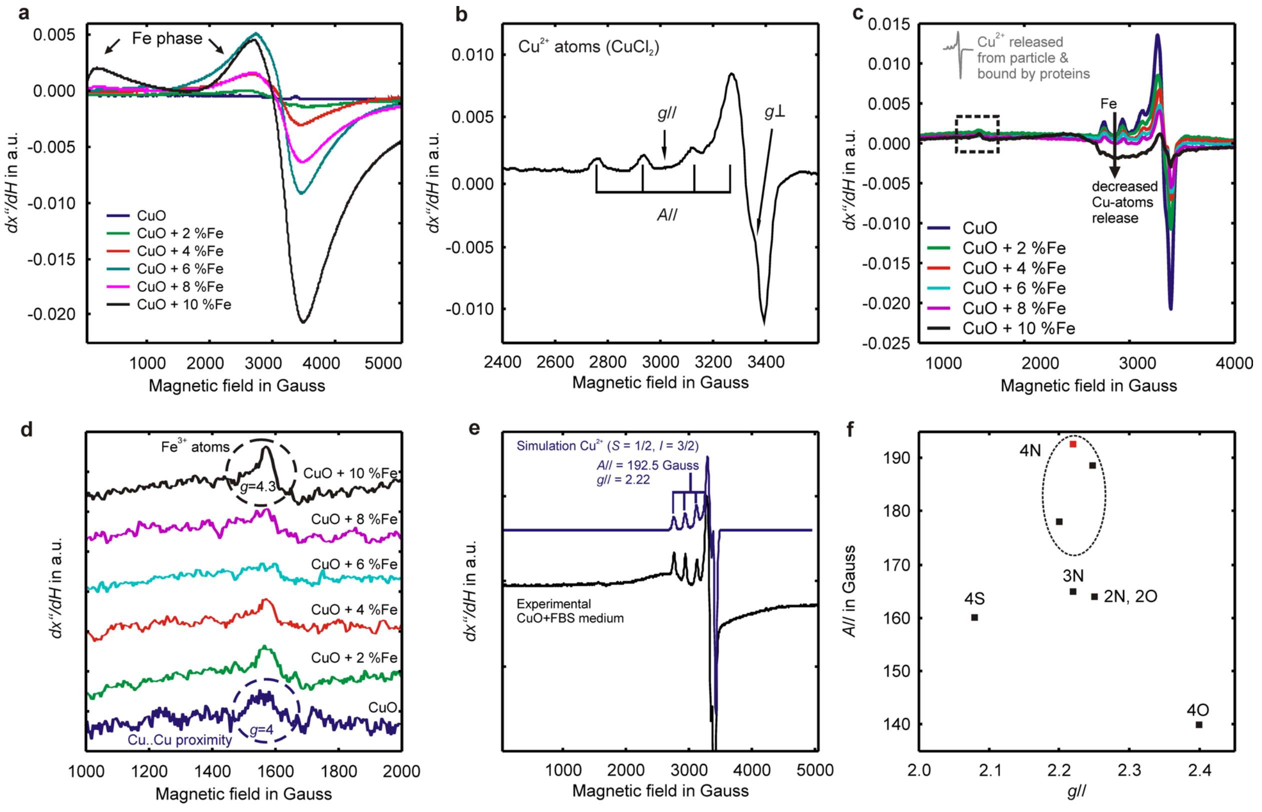

Figure S6. Transition from amino acid model solutions to biological environments. (a) The EPR spectra of as-prepared pure and Fe-doped CuO NPs show an increasing contribution of the Fe phase, indicating the existence of a small (< 2nm) superparamagnetic phase for the doped samples [13]. The Cu ions are exchange-coupled due to the high crystallinity of the CuO NPs. (b) In contrast, a typical EPR spectrum of externally-added (*via* $CuCl_2$) Cu^{2+} atoms ($S = 1/2$, $I = 3/2$) in the growth medium RPMI is presented. The spectrum consists of four hyperfine lines due to interaction of the Cu^{2+} spin $S = 1/2$ with the Cu nuclear spin $I = 3/2$ and serves as reference spectrum for tracing the binding of the Cu^{2+} ions released from the particles to the RPMI+10% FBS medium. (c) Spectra of particles in the growth medium show decreased dissolution (65% in pure CuO to 8% in 10% Fe-doped CuO, after 10 minutes) via doping. (d) Very low Fe^{3+} (<2% in 10% Fe-doped CuO) in the solution assigned through sharp EPR signal at $g = 4.3$. The broad peak at $g = 4$ is characteristic for Cu^{2+} release from CuO followed by protein complexation. (e) A theoretical simulation (blue line) was performed to calculate EPR parameters $A_{//} = 192.5$ Gauss and $g_{//} = 2.22$, matching with the experiment (black line). (f) According to Peisach and Blumberg [14], a correlation-plot of $g_{//}$ vs. $A_{//}$ values can be used to identify the types and number of atoms coordinating the Cu^{2+} centers [15]. In such plot, each set of Cu^{2+}-coordinating atoms is represented by a point on the plot. Accordingly, Cu^{2+} atoms are strongly coordinated by at least 2N atoms, *i.e.* 4N or 2N plus two strongly coordinating O-atoms in a distorted geometry, of the amino groups present in the growth medium RPMI.

Table S1. Model parameter used to describe the dissolution processes of pure, 1, 6 and 10 % Fe-doped CuO in 5 mM isoleucine, serine, threonine and valine solutions (Figure 1 and Figure S2).

Isoleucine

f_{Fe}	0	1	6	10
$d_{\mathrm{p,0}}$ in nm	11.8	12.0	10.3	10.7
$d_{\mathrm{Fe,s=1}}$ in nm	-	6.22	8.63	9.70
k_{Cu} in h^{-1} $mM^{-2.75}$	33.45	7.15	2.77	1.05
$k_{\#,s}$ in # nm^{-2}		2.92		
$c_{\mathrm{CuO.0}}$ in mM		2.04		
D in m^2 s^{-1}		1.59e-27		
MSE in mM^2		5.10e-03		

Serine

f_{Fe}	0	1	6	10
$d_{\mathrm{p,0}}$ in nm	11.8	12.0	10.3	10.7
$d_{\mathrm{Fe,s=1}}$ in nm	-	6.01	8.48	9.60
k_{Cu} in h^{-1} $mM^{-2.75}$	27.97	5.14	2.71	1.19
$k_{\#,s}$ in # nm^{-2}		3.76		
$c_{\mathrm{CuO.0}}$ in mM		2.04		
D in m^2 s^{-1}		4.30e-27		
MSE in mM^2		5.42e-03		

Threonine

f_{Fe}	0	1	6	10
$d_{\mathrm{p,0}}$ in nm	11.8	12.0	10.3	10.7
$d_{\mathrm{Fe,s=1}}$ in nm	-	6.76	8.92	9.89
k_{Cu} in h^{-1} $mM^{-2.75}$	73.86	13.13	8.11	2.46
$k_{\#,s}$ in # nm^{-2}		2.65		
$c_{\mathrm{CuO.0}}$ in mM		2.12		
D in m^2 s^{-1}		1.09e-26		
MSE in mM^2		8.86e-03		

Valine

f_{Fe}	0	1	6	10
$d_{\mathrm{p,0}}$ in nm	11.8	12.0	10.3	10.7
$d_{\mathrm{Fe,s=1}}$ in nm	-	6.35	8.71	9.75
k_{Cu} in h^{-1} $mM^{-2.75}$	46.30	9.59	6.61	1.57
$k_{\#,s}$ in # nm^{-2}		3.15		
$c_{\mathrm{CuO.0}}$ in mM		2.04		
D in m^2 s^{-1}		5.53e-27		
MSE in mM^2		4.90-03		

Table S2. Elemental composition of the 10 % Fe-doped CuO NP as-prepared sample. The atomic Fe/(Fe+Cu) ratio is 10.4 at%.

Element	Atomic number	Series	Normalized concentration in wt. %	Atomic concentration in at. %	Error (1 sigma) in wt. %
C	6	K-Series	6.31	16.25	1.20
O	8	K-Series	25.78	49.81	2.82
P	15	K-Series	0.95	0.95	0.07
Fe	26	K-Series	6.21	3.44	0.22
Cu	29	K-Series	60.75	29.55	1.67
Au	79	M-Series	0.00	0.00	0.00
		Sum Σ	100.00	100.00	

Table S3. Elemental composition of the 10 % Fe-doped CuO NP sample after dissolution for four weeks in 50 mM valine solution. The atomic Fe/(Fe+Cu) ratio is 87.3 at%.

Element	Atomic number	Series	Normalized concentration in wt. %	Atomic concentration in at. %	Error (1 sigma) in wt. %
C	6	K-Series	5.42	11.35	1.14
O	8	K-Series	39.24	61.66	4.36
Al	13	K-Series	0.26	0.25	0.05
Si	14	K-Series	1.49	1.34	0.09
P	15	K-Series	4.64	3.77	0.19
Fe	26	K-Series	41.96	18.89	1.15
Cu	29	K-Series	6.98	2.76	0.33
Au	79	M-Series	0.00	0.00	0.00
		Sum Σ	100.00	100.00	

Table S4. Single-crystal XRD, data collection and refinement details on Cu(II)-L-glutamine.

**Chemical formula**	$Cu[NH_2CO_2CH(CH_2)_2CONH_2]_2$
**M_r**	353.82
**Space group**	*C 2*
**Temperature (K)**	298
**a, b, c (Å); β (°);V (Å³)**	28.291(8), 5.0846(14), 9.420(3); 99.083(9); 1338.113
**Nreflections used for cell parameters**	1150
Z	4
**Data collection**	
**Radiation type, *λ* (Å)**	Mo K*α*, 0.71075
**Crystal size (mm)**	0.052 x 0.204 x 0.309
**Absorption correction**	SADABS
**μ (mm^{-1})**	1.66
**T_{min}, T_{max}**	0.6203, 0.7459
**No. of refl's all/ obs;**	13547/14542;
**independent all/ obs**	3141/3334
**R_{int} (obs/ all) (%)**	2.62/2.64
**θ_{max} (°); (sin $\theta/\lambda)_{max}$ (Å^{-1})**	29.58°; 0.6949
**Range of indices**	h = -38…38, k = -6…7, l = -12…11
**Refinement [a]**	
**Software**	JANA2006 [16]
**Start & reference model**	(CCDC 179785) [17]
**R, *wR*, *Gof* (all) (%)**	3.49, 3.40, 2.03
**R, *wR*, *Gof* [$F^2 > 3\sigma(F^2)$] (%)**	3.08, 3.35, 2.07
**Extinction correction**	none
**No. of parameters**	92
**$\Delta\rho_{max}$, $\Delta\rho_{min}$ (e Å^{-3})**	0.63, -0.75

[a] Refinements based on F values, R-factors: $R = \frac{\sum_i \left\| F_i(obs) \right| - \left| F_i(calc) \right\|}{\sum_i \left| F_i(obs) \right|}$, $R_w = \sqrt{\frac{\sum_i w_i \left(\left| F_i(obs) \right| - \left| F_i(calc) \right| \right)^2}{\sum_i w_i F_i(obs)^2}}$, $GOF = \sqrt{\frac{\sum_i w_i \left(\left| F_i(obs) \right| - \left| F_i(calc) \right| \right)^2}{n-p}}$,

with $w = \frac{1}{\sigma^2(F) + (0.01 \cdot F)^2}$

Table S5. To validate conservation of mass for the numerical solution scheme given in Supplementary Note 1, model radii after complete removal of copper $\boldsymbol{r}_{\infty,\mathbf{mod}}$ were compared with the theoretically expected values $\boldsymbol{r}_{\infty,\mathbf{theo}} = \sqrt[3]{\boldsymbol{f}_{\boldsymbol{Fe}} \boldsymbol{r}_\mathbf{0}^\mathbf{3}}$. A decreasing iron-copper ratio $\boldsymbol{f}_{\boldsymbol{Fe}}$ requires an increasing number of nodes $\boldsymbol{N}$ for an appropriate spatial resolution. For the fitting procedure in Supplementary Note 2, $\boldsymbol{N} = \mathbf{2500}$ was chosen.

$\boldsymbol{f}_{\boldsymbol{Fe}}$	$\boldsymbol{N}$	$\boldsymbol{r}_\mathbf{0}$	$\boldsymbol{r}_{\infty,\mathbf{theo}}$	$\boldsymbol{r}_{\infty,\mathbf{mod}}$	**Rel. error in %**
**0.25**	250	5	3.150	3.168	0.57
**0.10**	250	5	2.321	2.405	3.62
	1000	5	2.321	2.347	1.12
**0.06**	1000	5	1.957	2.001	2.25
**0.01**	1000	5	1.077	1.343	24.70
	2500	5	1.077	1.228	14.02
	5000	5	1.077	1.170	8.64

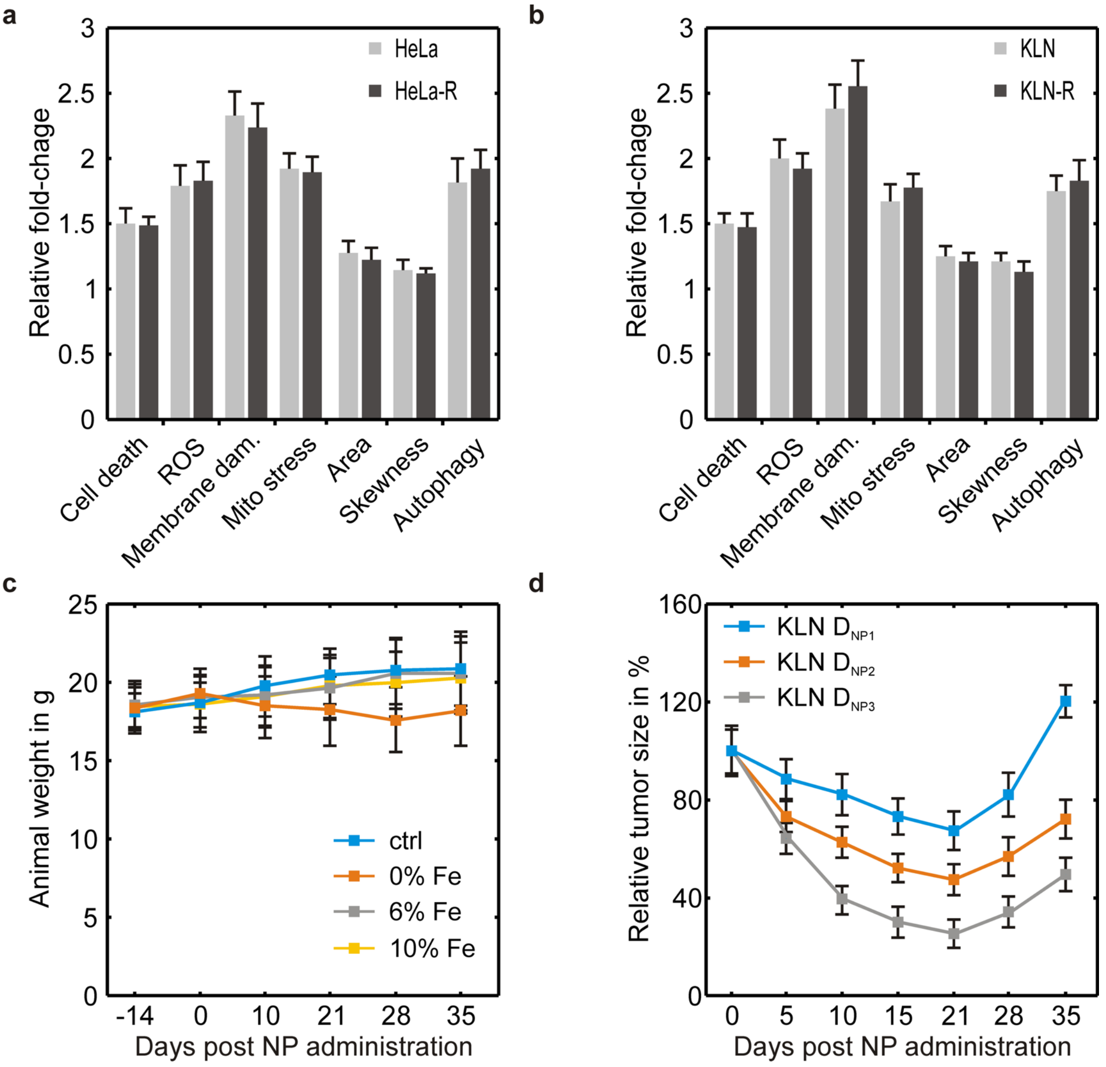

Figure S7. Histograms presenting the cellular parameters indicated for both wild-type and resistant tumor cells (cell-R) for (a) Hela and (b) KLN 205 cells exposed to 6% Fe-doped CuO at 12.5 µg/ml. (c) Animal weights expressed in grams for DBA/2 mice bearing KLN 205 cells treated with saline, CuO, 6% Fe-doped CuO or 10% Fe-doped CuO at 125 µg/mouse. (d) Relative luminescence signals for firefly luciferase-expressing KLN 205 cells grafted subcutaneously in syngeneic DBA/2 mice and treated with 6% Fe-doped CuO given at D_{NP1}: 125 µg/mouse, D_{NP2}: 175 µg/mouse and D_{NP3}: 225 µg/mouse. Data are presented as mean ± SD (n = 8).

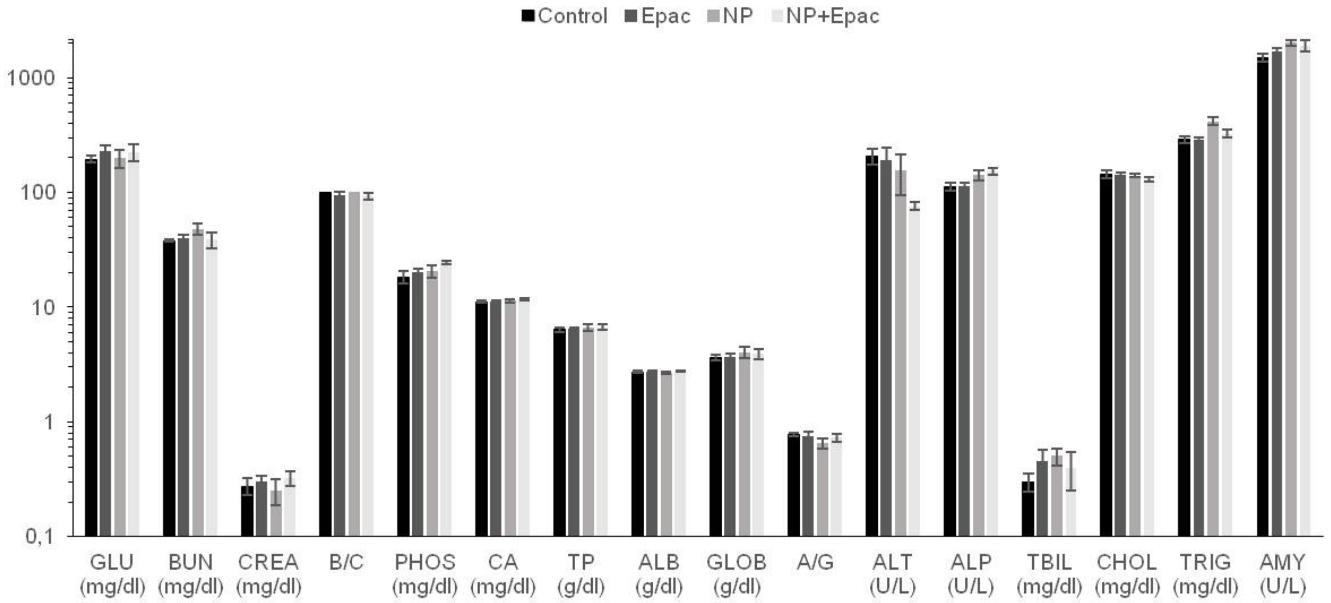

Figure S8. Blood biochemistry results from DBA/2 mice bearing KLN 205 tumors treated either with saline, epacadostat, 6% Fe-doped CuO (225 μg/mouse) with or without epacadostat. Data are presented with mean ± SD (n = 4).

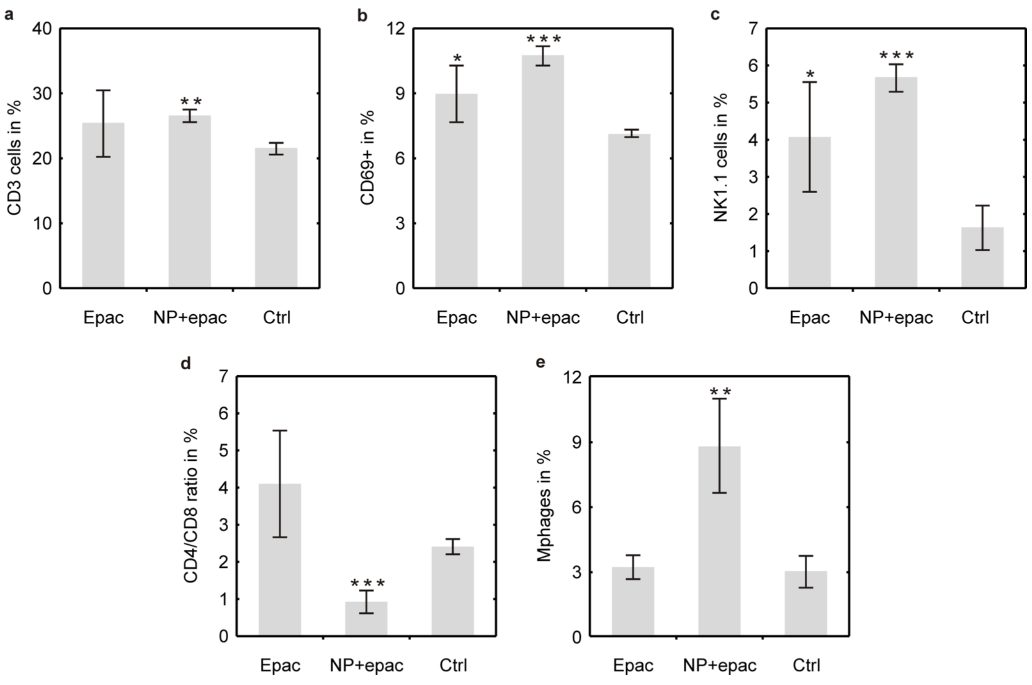

Figure S9. Quantification of imaging flow cytometry of tumor cells obtained from CT26 tumor-bearing Balb/c mice treated either with saline, epacadostat or 6% Fe-doped CuO + epacadostat and analyzed for (a) total CD^{3+} cells (total T cell population), (b) $CD69^{+}$ cells (T cell activation) or (c) $NK1.1^{+}$ cells (cytotoxic natural killer cells). Quantification of imaging flow cytometry of splenocytes obtained from CT26 tumor-bearing Balb/c mice treated either with saline, epacadostat or 6% Fe-doped CuO + epacadostat and analyzed for (d) CD4/CD8 or (f) macrophages. The data are presented as mean ± SD (n = 4). The number of asterisks indicate the level of significance, where: * = $p < 0.05$, ** = $p < 0.01$ and *** = $p < 0.001$.

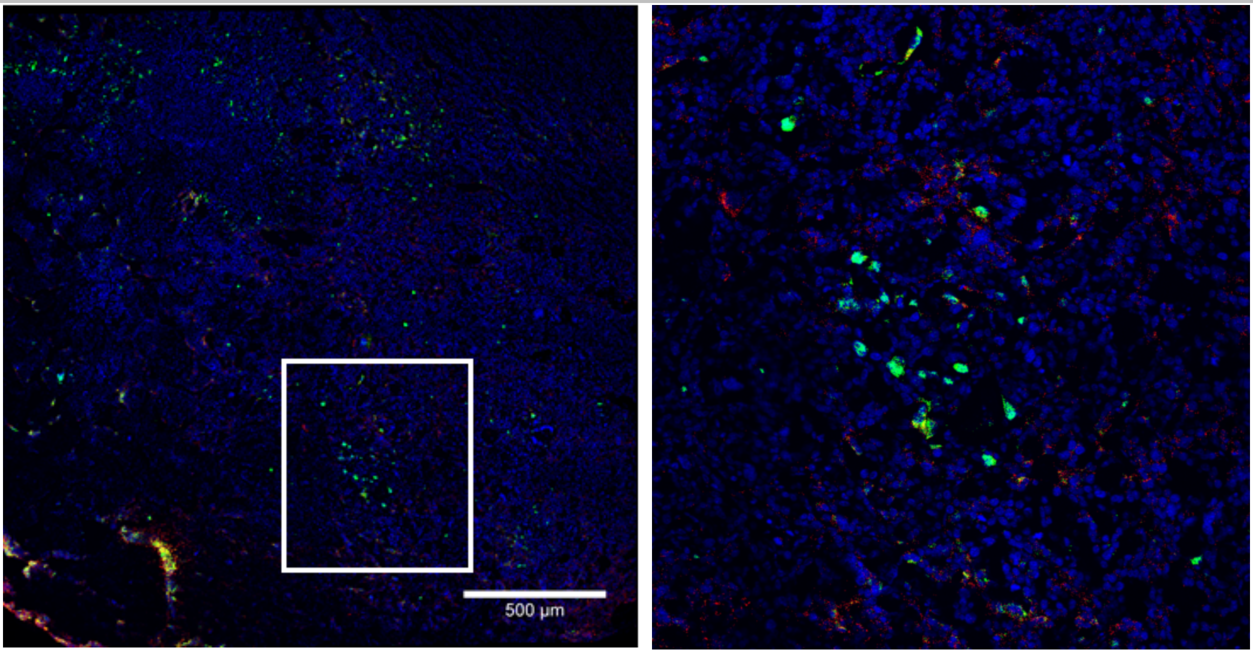

Figure S10. Representative immunofluorescence staining of wild-type CT26 tumor, treated with NPs and epacadostat, at 10 days following initial dosing. Tissues slices (10 μm cryotome slice) were stained against F4/80 (macrophages, green) and CD8 (cytotoxic T cells, red) and counterstained with Hoechst (nuclei, blue). The image on the right is a magnified image of the area indicated on the left.

References

[1] H. Naatz, S. Lin, R. Li, W. Jiang, Z. Ji, C. H. Chang, J. Köser, J. Thöming, T. Xia, A. E. Nel, L. Mädler, S. Pokhrel, ACS Nano **2017**, 11, 501-515.
[2] S. Pokhrel, A. E. Nel, L. Mädler, Acc. Chem. Res. **2013**, 46, 632-641.
[3] a) J. Watly, E. Simonovsky, N. Barbosa, M. Spodzieja, R. Wieczorek, S. Rodziewicz-Motowidlo, Y. Miller, H. Kozlowski, Inorg. Chem. **2015**, 54, 7692-7702; b) S. M. Leite, L. M. Lima, S. Gama, F. Mendes, M. Orio, I. Bento, A. Paulo, R. Delgado, O. Iranzo, Inorg. Chem. **2016**, 55, 11801-11814.
[4] R. Kayestha, Sumati, K. Hajela, FEBS Lett. **1995**, 368, 285-288.
[5] H. Naatz, S. Lin, R. Li, W. Jiang, Z. Ji, C. H. Chang, J. Köser, J. Thöming, T. Xia, A. E. Nel, L. Mädler, S. Pokhrel, ACS Nano **2017**, 11, 501-515.
[6] T. Sweijen, C. J. van Duijn, S. M. Hassanizadeh, Chem. Eng. Sci. **2017**, 172, 407-413.
[7] T. C. Illingworth, I. O. Golosnoy, J. Comput. Phys. **2005**, 209, 207-225.
[8] D. J. Nicolin, G. E. C. da Silva, R. M. M. Jorge, L. M. d. M. Jorge, J. Food Process Eng. **2018**, 41, e12693.
[9] S. Joshi, I. Ghosh, S. Pokhrel, L. Mädler, W. M. Nau, ACS Nano **2012**, 6, 5668-5679.
[10] A. E. Nel, L. Mädler, D. Velegol, T. Xia, E. M. Hoek, P. Somasundaran, F. Klaessig, V. Castranova, M. Thompson, Nat. Mater. **2009**, 8, 543-557.
[11] Y. Xu, M. A. A. Schoonen, American Mineralogist **2000**, 85, 543-556.
[12] Wellcome Sanger Institute, Massachusetts General Hospital Cancer Center, https://www.cancerrxgene.org/translation/Drug/133, accessed on 13.06.2019.
[13] J. T. N. Knijnenburg, E. Seristatidou, F. M. Hilty, F. Krumeich, Y. Deligiannakis, J. Phys. Chem. C **2014**, 118, 24072-24080.
[14] J. Peisach, W. E. Blumberg, Arch. Biochem. Biophys. **1974**, 165, 691-708.
[15] G. Grigoropoulou, K. C. Christoforidis, M. Louloudi, Y. Deligiannakis, Langmuir **2007**, 23, 10407-10418.
[16] V. Petříček, M. Dušek, L. Palatinus, Zeitschrift für Kristallographie - Crystalline Materials **2014**, 229.
[17] J. M. Schveigkardt, Alberto C. Rizzi, Oscar E. Piro, Eduardo E. Castellano, Ricardo Costa d. Santana, R. Calvo, Carlos D. Brondino, Eur. J. Inorg. Chem. **2002**, 2913-2919.

Author Contributions

H.N., S.P. and J.B. synthesized and/or characterized the materials. H.N. recorded and modelled the dissolution kinetics (Figure 2 and S1-S4). B.B.M, C.R.L and S.J.S designed, executed and analyzed the *in vitro* and *in vivo* cancer studies (Figure 3,4 and S7-S10). V.T and Y.D. contributed EPR studies (Figure S6). While all authors contributed to the text, L.M coordinated and supervised the project.
